# TAK1 Phosphorylates RASSF9 and Inhibits Esophageal Squamous Tumor Cell Proliferation by Targeting the RAS/MEK/ERK Axis

**DOI:** 10.1002/advs.202001575

**Published:** 2021-01-06

**Authors:** Hui Shi, Qianqian Ju, Yinting Mao, Yuejun Wang, Jie Ding, Xiaoyu Liu, Xin Tang, Cheng Sun

**Affiliations:** ^1^ Department of Cardiothoracic Surgery Nantong Key Laboratory of Translational Medicine in Cardiothoracic Diseases Nantong Clinical Medical Research Center of Cardiothoracic Disease Institution of Translational Medicine in Cardiothoracic Diseases Affiliated Hospital of Nantong University 20 Xisi Road Nantong 226001 China; ^2^ Key Laboratory for Neuroregeneration of Jiangsu Province and Ministry of Education Nantong University 19 Qixiu Road Nantong 226001 China

**Keywords:** cell proliferation, ERK, esophageal squamous tumor, MEK, RASSF9, TAK1

## Abstract

TGF‐*β*‐activated kinase 1 (TAK1), a serine/threonine kinase, is a key intermediate in several signaling pathways. However, its role in tumorigenesis is still not understood well. In this study, it is found that TAK1 expression decreases in esophageal tumor tissues and cell lines. In vitro experiments demonstrate that proliferation of esophageal tumor cells is enhanced by knockdown of TAK1 expression and attenuated by elevated expression of TAK1. Using a subcutaneous tumor model, these observations are confirmed in vivo. Based on the results from co‐immunoprecipitation coupled with mass spectrometry, Ras association domain family 9 (RASSF9) is identified as a downstream target of TAK1. TAK1 phosphorylates RASSF9 at S284, which leads to reduced RAS dimerization, thereby blocking RAF/MEK/ERK signal transduction. Clinical survey reveals that TAK1 expression is inversely correlated with survival in esophageal cancer patients. Taken together, the data reveal that TAK1‐mediated phosphorylation of RASSF9 at Ser284 negatively regulates esophageal tumor cell proliferation via inhibition of the RAS/MEK/ERK axis.

## Graphical abstract

TAK1 is decreased in esophageal squamous tumor cells, RASSF9 binds to RAS and causes RAS dimerization, which triggers RAF/MEK/ERK signal transduction and tumor cell proliferation. In the presence of TAK1, it phosphorylates RASSF9 at Serine284 and this modification disrupts RAS dimerization, leading to a blockade of downstream pathway and cell proliferation.

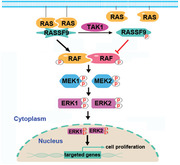

## 1 Introduction

Esophageal cancer, a malignancy in the tissues of the esophagus, is classified into two main sub‐types: esophageal squamous‐cell carcinoma (ESCC) and esophageal adenocarcinoma.^[^ ^1^ ^]^ Esophageal cancer was the eighth‐most common cancer globally with 456 000 new cases diagnosed in 2012^[^ ^2^ ^]^ and caused ≈400 000 deaths that year, up from 345 000 in 1990.^[^ ^2^, ^3^ ^]^ A major subtype of esophageal cancer, ESCC, accounts for approximately 90% of esophageal cancers and has been ranked as the fourth leading cause of cancer‐related mortality in China.^[^ ^4^ ^]^ Currently, there are no drugs that can cure esophageal cancer, and the treatment options are mainly limited to surgery, chemotherapy, and radiation therapy.^[^ ^4^ ^]^ Furthermore, high rates of tumor cell metastasis are frequently diagnosed in patients with resistance to these treatments.^[^ ^4^ ^]^ A clinical survey showed that the five‐year survival rate of patients diagnosed with esophageal cancer was around 13–18%.^[^ ^5^ ^]^ Therefore, there is an urgent need for in‐depth understanding of the mechanisms underlying cell growth and proliferation in esophageal cancer, as such knowledge may allow for development of more effective treatment strategies.

The TGF‐*β*‐activated kinase 1 (TAK1) protein, encoded by the Mitogen‐activated protein kinase kinase kinase 7 (Map3k7) gene, is a serine/threonine kinase that mediates proinflammatory cytokine‐related signaling pathways and plays multiple roles in cell fate regulation.^[^ ^6^, ^7^ ^]^ As a hub protein between membrane receptors and nuclear transcription factors, TAK1 is involved in reprogramming transcription in cancer cells toward proliferation, survival, and resistance to chemotherapy.^[^ ^8^ ^]^ TAK1 promotes tumorigenesis in several types of tumors, including breast cancer, colon cancer, and melanoma,^[^ ^9^, ^10^ ^]^ and consistent with its role in tumorigenesis, inhibition of TAK1 induces tumor cell apoptosis and reduces its metastatic capacity.^[^ ^11^, ^12^, ^13^ ^]^ Interestingly, however, several other studies have found that TAK1 appears to function as a tumor suppressor in liver and prostate carcinogenesis^[^ ^14^, ^15^, ^16^ ^]^ and conditional TAK1 knockout in liver parenchymal cells was reported to induce liver carcinogenesis.^[^ ^15^, ^17^ ^]^ Therefore, the role of TAK1 in carcinogenesis remains debatable.

The Ras association domain family (RASSF) consists of two subclasses of proteins: C‐RASSF and N‐RASSF. C‐RASSF proteins (RASSF1‐6) are characterized by a C‐terminal coiled‐coil motif known as the Salvador/RASSF/Hippo domain, whereas N‐RASSF proteins (RASSF7‐10) lack this motif.^[^ ^18^ ^]^ The Ras proteins belong to a family of small guanine triphosphatases (GTPase), which integrate signals from a variety of upstream sources and finely regulate cellular physiology.^[^ ^19^, ^20^, ^21^ ^]^ RAS activation promotes cell motility and lymph mode metastasis, leading to poorer survival in ESCC patients.^[^ ^22^ ^]^ Recently, RAS guanyl releasing protein 3 (RasGRP3), a Ras activator, was validated as an activator of the Notch pathway, thus leading to the development of ESCC.^[^ ^23^ ^]^ RASSFs play an essential role in cell growth and behavior through interaction with the RAS proteins.^[^ ^24^ ^]^ Several studies have shown that C‐RASSFs are suppressed in human cancers and inhibition of individual C‐RASSFs promotes tumor progression.^[^ ^18^, ^25^, ^26^ ^]^ Compared to the C‐RASSFs, investigations into the role of N‐RASSFs in tumorigenesis are limited.

In this study, we examined whether and how TAK1 affects esophageal squamous tumor cell proliferation. Our results showed that TAK1 is a negative regulator of tumor cell proliferation and phosphorylates RASSF9 at serine 284. Our results further revealed that TAK1‐induced phosphorylation of RASSF9 impairs RAS dimerization and leads to a shutdown of downstream signal transduction involving the RAF/MEK/ERK axis. Collectively, our data imply that TAK1 and its substrate RASSF9 represent potential diagnostic markers and/or drug targets for clinical treatment of esophageal cancer.

## 2 Results

### 2.1 TAK1 Shows Reduced Expression in Esophageal Squamous Tumor Tissues

**Figure 1:**
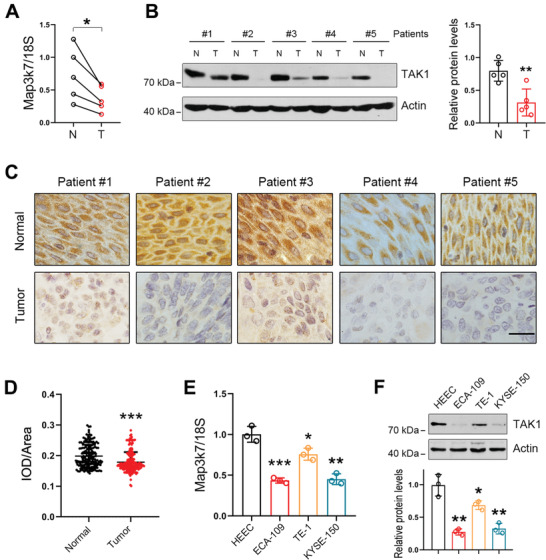
TAK1 shows reduced expression in esophageal squamous tumor tissues. A) mRNA levels of TAK1 in normal and esophageal squamous tumor tissues. *n* = 5. B) Protein levels of TAK1 in normal and esophageal squamous tumor tissues. *n* = 5. C) Immunohistochemical analysis of TAK1 expression in normal and esophageal squamous tumor tissues. Scale bar = 20 µm. D) Immunohistochemical analysis for TAK1 expression in large‐scale clinical samples including normal and esophageal squamous tumor tissues. IOD: integral optical density. *n* = 193. E) mRNA levels of TAK1 in HEEC and esophageal squamous tumor cell lines (ECA‐109, TE‐1, and KYSE‐150) (*n* = 3 biologically independent replicates per group). F) Protein levels of TAK1 in HEEC, ECA‐109, TE‐1, and KYSE‐150 cells. Representative blots were shown (*n* = 3 biologically independent replicates per group). mRNA levels of TAK1 were analyzed by qRT‐PCR and 18S was used for normalization of the gene expression. Protein levels of TAK1 were measured using western blot analysis and Actin was used as a loading control. Data are presented as mean ± standard deviation (SD; error bars). Statistical significance was tested by two‐tailed unpaired Student's *t*‐test. *p < 0.05, **p < 0.01, and ***p < 0.001.

To examine the potential role of TAK1 in ESCC progression, we first analyzed TAK1 expression both at mRNA and protein levels in clinical samples from ESCC patients. As shown in **Figure** **1**A, the mRNA level of TAK1 was markedly decreased in ESCC tissues compared to that in the adjacent normal tissues. A similar pattern was also observed for TAK1 protein expression (Figure 1B). Immunohistochemical staining revealed low expression level of TAK1 in tumor tissues (Figure 1C). Examination using large‐scale clinical samples also showed that TAK1 expression was decreased in esophageal squamous tumors (Figure 1D). Furthermore, we analyzed TAK1 expression in several esophageal squamous tumor cell lines, including ECA‐109, TE‐1, and KYSE‐150. As observed in the ESCC tissues, we found that TAK1 expression was markedly reduced in these cell lines (Figure 1E,F). Collectively, these data show that TAK1 expression levels are reduced in esophageal squamous tumors, which strongly implies that TAK1 is likely involved in esophageal squamous tumor progression.

### 2.2 TAK1 Negatively Regulates Esophageal Squamous Tumor Cell Proliferation

**Figure 2:**
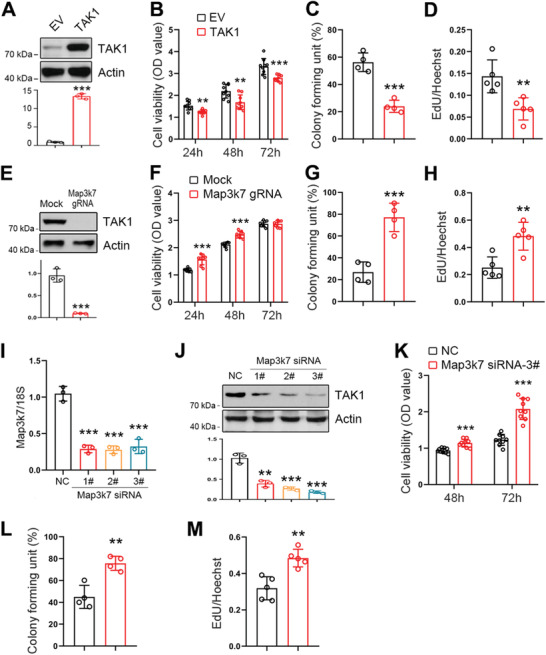
TAK1 negatively regulates esophageal squamous tumor cell proliferation. A) Increased expression of TAK1 in ECA‐109 cells transfected with plasmid expressing TAK1. B–D) Increased expression of TAK1 in ECA‐109 cells inhibits cell viability (B; *n* = 9 biologically independent replicates per group), colony formation (C; *n* = 4 biologically independent replicates per group), and EdU incorporation (D; *n* = 5 biologically independent replicates per group). E) TAK1 expression was decreased by Map3k7 gRNA in ECA‐109 cells. Protein level was detected by western blotting. Actin was used as a loading control. F–H) Reduced expression of TAK1 stimulates cell viability (F; *n* = 9 biologically independent replicates per group), colony formation (G; *n* = 4 biologically independent replicates per group), and EdU incorporation (H; *n* = 5 biologically independent replicates per group). I,J) ECA‐109 cells were transfected with siRNAs targeting Map3k7. TAK1 expression was measured using qRT‐PCR (I; *n* = 3 biologically independent replicates per group) and western blotting. 18S was used as an internal control for normalization of gene expression and Actin was used as a loading control. K–M) Knockdown of TAK1 promotes cell viability (K; *n* = 9 biologically independent replicates per group), colony formation (L; *n* = 4 biologically independent replicates per group), and EdU incorporation (M; *n* = 5 biologically independent replicates per group) in ECA‐109 cells. Sample size for data in (A, E, J) is *n* = 3 biologically independent replicates per group and representative blots were shown. Data are presented as mean ± SD (error bars). Statistical significance was tested by two‐tailed unpaired Student *t*‐test. *p < 0.05, **p < 0.01, and ***p < 0.001.

We next sought to determine whether TAK1 was involved in esophageal squamous tumor progression. To that end, we first increased TAK1 expression in ECA‐109 cells by transfecting the cells with a TAK1 expression plasmid. Western blot analysis confirmed that TAK1 expression was successfully enhanced by the transfection (**Figure** **2**A). We found that elevated TAK1 expression caused significant reduction in cell viability (Figure 2B). Cell proliferation was also retarded by elevated TAK1 expression based on the results from cell colony formation and EdU incorporation assays (Figure 2C,D and Figure S1A,B, Supporting Information). Moreover, we also observed that TAK1 reduced cell viability in a dose‐dependent manner (Figure S1C,D, Supporting Information). We similarly analyzed the effect of TAK1 overexpression on cell proliferation in two other esophageal squamous tumor cell lines (TE‐1 and KYSE‐150), and found that TAK1 overexpression caused reduction in cell viability, colony formation, and EdU incorporation in these two cell lines (Figures S2A–F and S3A–F, Supporting Information).

To further ascertain the effect of TAK1 in esophageal squamous tumor progression, we downregulated TAK1 expression in ECA‐109 cells using CRISPR/Cas9‐mediated genome editing. Our results showed that the small guide mRNA (gRNA) effectively inhibited TAK1 expression in ECA‐109 cells (Figure 2E) and TAK1 deficiency resulted in increased cell viability (Figure 2F). Cell proliferation was also enhanced by the gRNA against Map3k7 (Figure 2G,H and Figure S4A,B, Supporting Information). In addition, we used small interference RNAs (siRNAs) to downregulate TAK1 expression (Figure 2I,J). Out of the various siRNAs tested, siRNA 3# exhibited the best knockdown efficiency, and thus, it was chosen for subsequent experiments. Consistent with the observations of gRNA‐mediated downregulation of TAK1, siRNA‐induced TAK1 knockdown also potentiated cell viability, colony formation, and EdU incorporation (Figure 2K–M and Figure S4C,D, Supporting Information). Similarly, TAK1 knockdown induced upregulation of cell viability, colony formation and, EdU incorporation in TE‐1 and KYSE‐150 cells (Figures S5A–E and S6A–E, Supporting Information). Furthermore, we examined whether TAK1 affects cell apoptosis. In ECA‐109 cells, TAK1 overexpression stimulated cell apoptosis and TAK1 knockdown reduced cell apoptosis (Figure S7A–F, Supporting Information). The observed cell apoptosis induced by TAK1 may account for the decrease in cell proliferation. However, the EdU incorporation results, together with cell colony formation data, still suggest that TAK1 has a negative impact on cell proliferation. Collectively, these results indicate that TAK1 is a negative regulator of cell proliferation in cultured esophageal squamous tumor cells.

### 2.3 TAK1 Inhibits Esophageal Squamous Tumor Proliferation In Vivo

**Figure 3:**
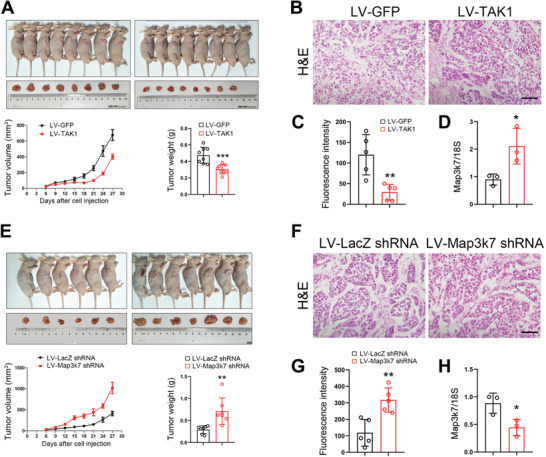
TAK1 negatively regulates esophageal squamous tumor cell growth in vivo. A) ECA‐109 cells were transduced with lentivirus expressing TAK1 (LV‐TAK1) or GFP (LV‐GFP). After selection using puromycin, the cells were implanted into nude mice. Tumor volume and weight were reduced with the LV‐TAK1 transduced cells. *n* = 8. B) H&E staining of the tumors transduced with LV‐TAK1. Scale bar = 200 µm. C) Quantitative analysis of immunostaining of Ki67 in the tumors transduced with LV‐TAK1. *n* = 5. D) qRT‐PCR analysis of TAK1 expression in LV‐TAK1 transduced tumors. *n* = 3. E) ECA‐109 cells were transduced with lentivirus bearing Map3k7 shRNA (LV‐Map3k7 shRNA) or LacZ shRNA (LV‐LacZ shRNA). After selection using puromycin, the cells were implanted into nude mice. Tumor volume and weight were improved with the LV‐Map3k7 shRNA transduced cells. *n* = 6, 7. F) H&E staining of the tumors transduced with LV‐Map3k7 shRNA. Scale bar = 200 µm. G) Quantitative analysis of Ki67 staining of the tumors transduced with LV‐Map3k7 shRNA. *n* = 5. H) qRT‐PCR analysis of TAK1 expression in tumors transduced with LV‐Map3k7 shRNA. *n* = 3. Data are presented as mean ± SD (error bars). Statistical significance was tested by two‐tailed unpaired Student *t*‐test. *p < 0.05, **p < 0.01, and ***p < 0.001.

We next sought to determine whether TAK1 was a negative regulator tumor cell proliferation in vivo. Toward that, we first transduced ECA‐109 cells with lentivirus expressing TAK1 (LV‐TAK1) and then transplanted these cells into nude mice. Our results showed that elevated expression of TAK1 induced by LV‐TAK1 inhibited cell growth, as evidenced by a reduction in tumor volume and weight (**Figure** **3**A). The morphology of these tumors was examined by hematoxylin & eosin (H&E) staining (Figure 3B). Cell proliferation was analyzed by Ki67 immunostaining and results showed that TAK1 markedly attenuated cell proliferation (Figure 3C and Figure S8A, Supporting Information). TAK1 expression in LV‐TAK1 transduced tumors was confirmed using qRT‐PCR (Figure 3D). In contrast, TAK1 knockdown by LV‐Map3k7 shRNA promoted tumor cell growth (Figure 3E). H&E staining is shown in Figure 3F. Cell proliferation in these tumors was enhanced following TAK1 knockdown (Figure 3G and Figure S8B, Supporting Information). TAK1 expression was reduced by LV‐Map3k7 shRNA (Figure 3H). Moreover, we also performed these in vivo experiments by using TE‐1 cells. Our data showed that tumor volume and weight were enhanced by TAK1 knockdown (Figure S9A–E, Supporting Information). Of note, the Ki67 immunostaining data further confirmed the negative regulation of TAK1 on cell proliferation, although TAK1 induces cell apoptosis (Figure S7, Supporting Information). These data further indicate that TAK1 is a negative regulator of cell growth and proliferation in esophageal squamous tumor.

### 2.4 TAK1 Binds to RASSF9 and Phosphorylates RASSF9 at Serine 284

**Figure 4:**
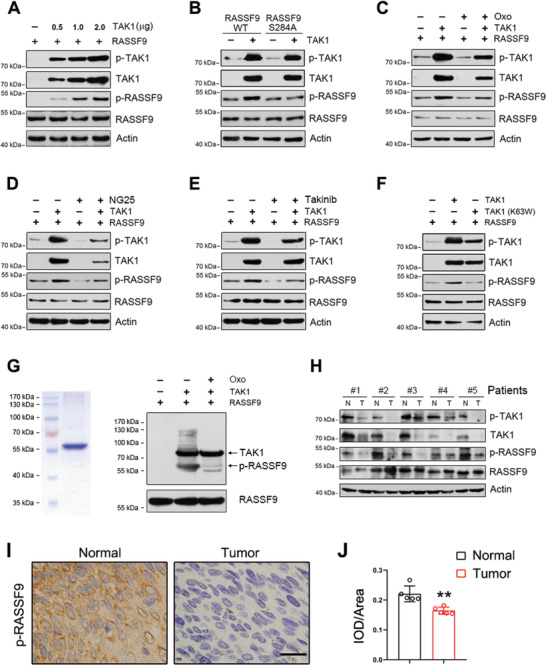
TAK1 phosphorylates RASSF9 in esophageal squamous tumor cells. A) TAK1 phosphorylates RASSF9 at Ser284. ECA‐109 cells were co‐transfected with plasmids expressing RASSF9 or TAK1. Total cell lysates were prepared and subjected to western blot analysis. B) TAK1 failed to phosphorylate RASSF9^S284A^. ECA‐109 cells were co‐transfected with the plasmids containing wildtype RASSF9, the S284A mutant of RASSF9 (RASSF9^S284A^), or TAK1 as indicated. C–E) Inhibition of TAK1 reduced RASSF9 phosphorylation. ECA‐109 cells were co‐transfected with the plasmids expressing RASSF9 or TAK1. TAK1 inhibitor (5Z)‐7‐Oxozeaenol (Oxo) (C), or NG25 (D), or Takinib (E) was added in culture medium 6 h post‐transfection, and the cells were cultured for an additional 18 h. Oxo: 10 µm; NG25: 10 µm; Takinib: 10 µm. F) Dominant negative TAK1 (K63W) failed to phosphorylate RASSF9 at Ser284. ECA‐109 cells were co‐transfected with plasmids expressing RASSF9 and TAK1 or TAK1 (K63W) as indicated. G) In vitro kinase assay showing TAK1 phosphorylates RASSF9 at Ser284. Recombinant human RASSF9 fused with His tag was confirmed by Coomassie blue staining. To analyze TAK1‐mediated phosphorylation, RASSF9 was incubated with active recombinant TAK1 in the presence or absence of Oxo (10 µm). The samples were separated by SDS‐PAGE and western blotting was used to detect p‐RASSF9, RASSF9, and TAK1. H) Decreased expression of TAK1 in esophageal tumor tissues correlates with reduced phospho‐RASSF9 (p‐RASSF9). Protein levels were detected by western blotting. Actin was used as a loading control. *n* = 5. I) Immunohistochemistry staining of p‐RASSF9 in normal and esophageal tumor tissues. Representative images were shown. *n* = 5. Scale bar = 20 µm. J) Quantitative analysis of p‐RASSF9 staining as shown in (I). IOD: integral optical density. Sample size for data in (A–F) is *n* = 3 biologically independent replicates per group and representative blots were shown. Data are presented as mean ± SD (error bars). Statistical significance was tested by two‐tailed unpaired Student *t*‐test. **p < 0.01.

To determine the mechanisms underlying TAK1‐mediated suppression of cell proliferation in esophageal tumor, we performed co‐immunoprecipitation coupled with mass spectrometry analysis to identify the downstream targets of TAK1. Mass spectrometric data revealed that 24 proteins were phosphorylated in the co‐immunoprecipitation complex obtained using anti‐TAK1 antibody (Supporting Information 1). Of these proteins, RASSF9 is known to play critical roles in tumor cell physiology.^[^ ^24^, ^25^, ^27^ ^]^ Based on the mass spectra data (Figure S10A,B, Supporting Information), serine residue at 284 (S284) in RASSF9 was found to be phosphorylated. Interestingly, this site is highly conserved among RASSF9s from different species (Figure S10C, Supporting Information). However, as antibodies against RASSF9 and RASSF9 phosphorylated at S284 (p‐RASSF9) were not available, we generated these antibodies. Immunoblot data using these antibodies revealed the molecular weight of RASSF9 as ≈50 kDa (**Figure** **4**A). Further, p‐RASSF9 was increased by TAK1 in a dose‐dependent manner (Figure 4A). The interaction between TAK1 and RASSF9 was further ascertained by co‐immunoprecipitation and pull‐down assays (Figure S10D,E, Supporting Information). To confirm that TAK1 phosphorylates S284 in RASSF9, we constructed a mutant form of RASSF9 (S284A) wherein serine 284 was replaced with an alanine. As shown in Figure 4B, TAK1 induced an increase in p‐RASSF9 in the cells transfected with wildtype RASSF9; however, TAK1 had no effect on p‐RASSF9 in the cells transfected with RASSF9 S284A. We also used (5Z)‐7‐oxozeaenol (Oxo), a TAK1 inhibitor, to further confirm that TAK1 phosphorylates S284 in RASSF9. Our results showed that TAK1 caused phosphorylation of RASSF9 and treatment with Oxo attenuated this modification (Figure 4C). To confirm this finding, two other TAK1 inhibitors, NG25 and Takinib, were also tested. The results showed that p‐RASSF9 induced by TAK1 was largely decreased in the presence of these inhibitors (Figure 4D,E). In addition, TAK1‐mediated phosphorylation of RASSF9 at S284 could not be recapitulated by a dominant negative form of TAK1 (Figure 4F), in which lysine 63 was mutated into tryptophan (K63W). Furthermore, in vitro kinase assay also revealed that the S284 in RASSF9 was phosphorylated by TAK1 (Figure 4G). Similar to the decline in p‐TAK1 and TAK1 levels in the human samples, the p‐RASSF9 level was decreased in esophageal squamous tumor tissues (Figure 4H). Immunohistochemical analysis also showed that p‐RASSF9 was reduced in tumors (Figure 4I,J). Taken together, we conclude that TAK1 phosphorylates RASSF9 at S284 and thus RASSF9 is a downstream target of TAK1.

### 2.5 TAK1 Inhibits Esophageal Squamous Tumor Cell Proliferation by Phosphorylating RASSF9

**Figure 5:**
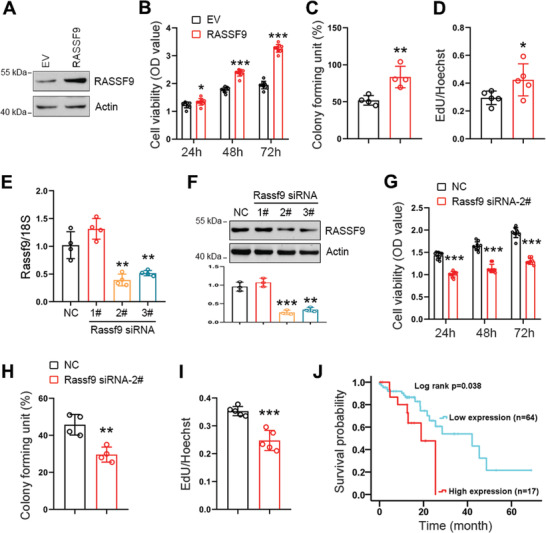
RASSF9 positively regulates esophageal squamous tumor cell proliferation. A) ECA‐109 cells were transfected with plasmid expressing RASSF9. Western blot analysis showing RASSF9 was increased following the transfection. Actin was used as a loading control. Representative blots were shown (*n* = 3 biologically independent replicates per group). B–D) Elevated expression of RASSF9 stimulates cell viability (B; *n* = 9 biologically independent replicates per group), colony formation (C; *n* = 4 biologically independent replicates per group), and EdU incorporation (D; *n* = 5 biologically independent replicates per group) in ECA‐109 cells. E) ECA‐109 cells were transfected with siRNAs targeting Rassf9. Rassf9 siRNAs decreased mRNA levels of Rassf9 (*n* = 3 biologically independent replicates per group). Gene expression was analyzed by qRT‐PCR and 18S was used for normalization of the gene expression. F) Protein level of RASSF9 was reduced by Rassf9 siRNAs in ECA‐109 cells (*n* = 3 biologically independent replicates per group). Protein level was analyzed by western blotting and Actin was used a loading control. Representative blots were shown (*n* = 3 biologically independent replicates per group). G–I) Knockdown of RASSF9 decreases cell viability (G; *n* = 9 biologically independent replicates per group), colony formation (H; *n* = 4 biologically independent replicates per group), and EdU incorporation (I; *n* = 5 biologically independent replicates per group) in ECA‐109 cells. J) TCGA database showing that RASSF9 expression negatively correlates with survival time in ESCC patients. Data are presented as mean ± SD (error bars). Statistical significance was tested by two‐tailed unpaired Student *t*‐test (B–I) or log‐rank (Kaplan–Meier) test (J). *p < 0.05, **p < 0.01, and ***p < 0.001.

We next sought to determine whether TAK1 regulates tumor cell proliferation through RASSF9. Therefore, we examined the effect of RASSF9 on cell proliferation. To that end, we transfected ECA‐109 cells with a plasmid expressing RASSF9 and subsequently analyzed cell growth and proliferation. Our results showed that while RASSF9 overexpression stimulated cell viability, colony formation, and EdU incorporation (**Figure** **5**A–D and Figure S11A,B, Supporting Information), the knockdown of RASSF9 delayed cell growth and proliferation (Figure 5E–I and Figure S11C,D, Supporting Information). Human TCGA database revealed that high expression of RASSF9 was correlated with poorer prognosis in ESCC patients; and conversely, low expression of RASSF9 was associated with longer survival (Figure 5J).

**Figure 6:**
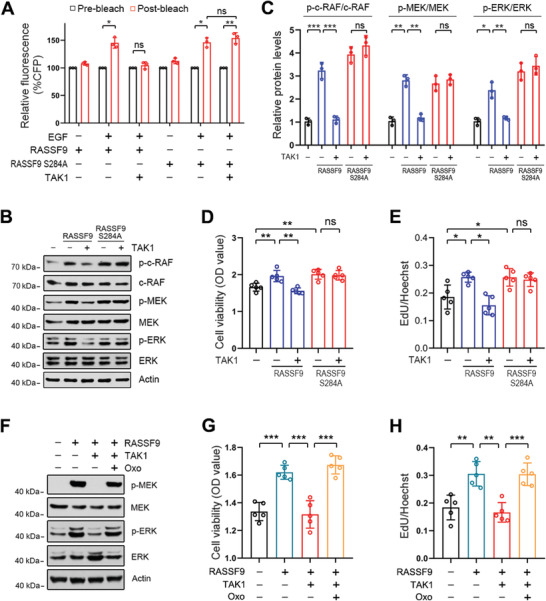
TAK1 inhibits RASSF9‐induced signal transduction through the RAS/RAF/MEK/ERK axis. A) TAK1 inhibits RASSF9‐mediated RAS dimerization. ECA‐109 cells were co‐transfected with plasmids expressing CFP‐KRAS, YFP‐KRAS, RASSF9, RASSF9^S284A^, and TAK1 as indicated. CFP emission was assayed by FRET (*n* = 3 biologically independent replicates per group). B) TAK1 blocks signal transduction through the RAF/MEK/ERK axis induced by RASSF9. ECA‐109 cells were co‐transfected with plasmids containing RASSF9, RASSF9^S284A^, or TAK1. Signal transduction through the RAF/MEK/ERK axis was analyzed by western blotting using antibodies as indicated. Representative blots were shown (*n* = 3 biologically independent replicates per group). C) Quantitative analysis of the western blot data shown in (B). D) TAK1 has no effects on cell viability induced by RASSF9^S284A^. ECA‐109 cells were co‐transfected with plasmids expressing TAK1, RASSF9, or RASSF9^S284A^. 36 h post‐transfection, cell viability was analyzed by CCK8 (*n* = 5 biologically independent replicates per group). E) TAK1 fails to inhibit cell proliferation induced by RASSF9^S284A^. Cell treatments were described in (D). 36 h post‐transfection, cell proliferation was evaluated by EdU incorporation assay (*n* = 5 biologically independent replicates per group). F) Inhibition of TAK1 ameliorates RASSF9 downstream signal transduction. ECA‐109 cells were transfected with plasmids expressing RASSF9 or TAK1 as indicated. 12 h post‐transfection, the cells were treated with 10 µm Oxo for additional 24 h. Representative blots were shown (*n* = 3 biologically independent replicates per group). G) TAK1 inhibition recues decreased cell viability induced by TAK1. Cell treatments were described in (F). Cell viability was assayed by CCK8 (*n* = 5 biologically independent replicates per group). H) TAK1 inhibition recues decreased cell proliferation induced by TAK1. Cell treatments were described in (F). Cell proliferation was analyzed by EdU incorporation assay (*n* = 5 biologically independent replicates per group). Protein levels were measured by western blot analysis. Actin was used as a loading control. Data are presented as mean ± SD (error bars). Statistical significance was tested by two‐tailed one‐way ANOVA test. *p < 0.05, **p < 0.01, and ***p < 0.001. ns means no significance.

Given that RASSF9 contains a RAS association (RA) domain, we reasoned that RASSF9 could be a positive regulator of the RAS signaling pathway by inducing RAS dimerization. We thus employed a cell‐based FRET system using CFP (donor) and YFP (acceptor) fusions of KRAS to evaluate the effect of RASSF9 on KRAS dimerization.^[^ ^19^ ^]^ We found that the CFP signal increased significantly after YFP bleaching and this increase was further strengthened in the presence of wildtype RASSF9 or the mutant of RASSF9 (**Figure** **6**A). TAK1 markedly abolished CFP signal enhancement in the presence of wildtype RASSF9 but had no effect in the presence of the mutant of RASSF9 (Figure 6A). It is well documented that the RAS/RAF/MEK/ERK signaling pathway is a pivotal cascade for driving tumor cell proliferation.^[^ ^28^ ^]^ Therefore, we next examined the effect of RASSF9 on RAF/MEK/ERK signal transduction. We found that RASSF9 stimulated the RAF/MEK/ERK axis, as evidenced by the increased levels of phosphorylated RAF, MEK, and ERK (p‐RAF, p‐MEK, and p‐ERK); however, these stimulations were greatly reduced in the presence of TAK1 (Figure 6B,C). While the mutant of RASSF9 (S284A) similarly stimulated the RAF/MEK/ERK axis, its effect was not altered by TAK1 (Figure 6B,C). Accordingly, the increases in cell viability and proliferation induced by RASSF9 were reduced by forced expression of TAK1; however, these increases induced by RASSF9 S284A were not altered by TAK1 (Figure 6D,E and Figure S12, Supporting Information). Subcutaneous tumorigenesis study further confirmed that TAK1 lost the inhibitory effect on RASSF9 S284A induced tumor cell growth (Figure S13, Supporting Information). Moreover, TAK1‐mediated inhibition in RASSF9 induced downstream signal transduction and cell proliferation were largely rescued by Oxo‐mediated inhibition of TAK1 (Figure 6F–H and Figure S14, Supporting Information).

**Figure 7:**
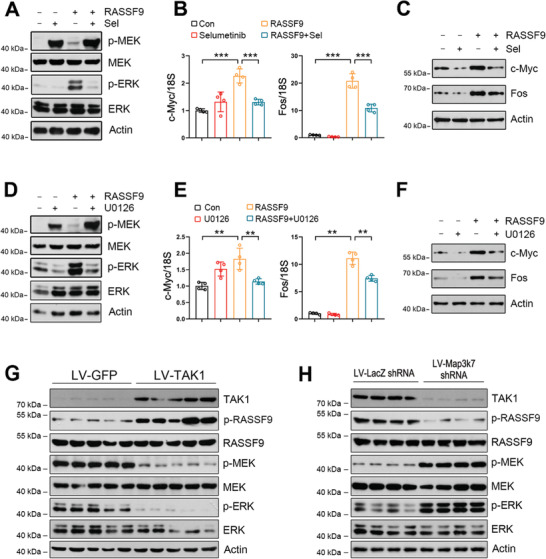
MEK inhibition counteracts RASSF9‐induced ERK activation and oncogene expression. A) MEK inhibition by selumetinib reduces RASSF9‐induced ERK activation. B–C) MEK inhibition by selumetinib decreases the expression of c‐Myc and Fos at B) mRNA and C) protein levels. ECA‐109 cells were transfected with plasmid carrying RASSF9. The cells were treated with 10 µm of selumetinib 12 h post transfection, for additional 24 h. D) MEK inhibition using U0126 abolishes RASSF9‐induced ERK activation. E,F) U0126 blocks RASSF9‐induced the expression of c‐Myc and Fos at E) mRNA and F) protein levels. Cell treatments were similar to the procedures in (A–C) except that 10 µm U0126 was used instead of 10 µm selumetinib. G,H) Protein levels of TAK1, p‐RASSF9, RASSF9, p‐MEK, MEK, p‐ERK, ERK in the transplanted tumors in nude mice. The transplantation procedures were described in Figure 3. *n* = 5. Protein and mRNA levels were analyzed by western blot analysis and qRT‐PCR, respectively. Actin was used as a loading control. 18S was used for normalization of the gene expression in qRT‐PCR. Sel: selumetinib. Sample size for data in (A, C, D, F) is *n* = 3 biologically independent replicates per group and representative blots were shown. Sample size for data in (B, E) is *n* = 4 biologically independent replicates per group. Data are presented as mean ± SD (error bars). Statistical significance was tested by two‐tailed one‐way ANOVA test. **p < 0.01 and ***p < 0.001.

We next chose a pharmacological intervention strategy to confirm these findings. As shown in **Figure** **7**A, p‐MEK was stimulated by RASSF9. Treatment with the MEK inhibitor, selumetinib, increased p‐MEK with or without RASSF9 (Figure 7A). However, p‐ERK was inhibited by selumetinib in the presence or absence of RASSF9 (Figure 7A). We also examined the expression levels of Fos and c‐Myc, which are the two main genes activated by the RAF/MEK/ERK signaling pathway.^[^ ^29^, ^30^ ^]^ We found that the mRNA and protein levels of c‐Myc and Fos were enhanced by RASSF9, but this enhancement was significantly counteracted by selumetinib treatment (Figure 7B,C). Similarly, p‐MEK was increased by U0126. RASSF9 induced an increase in p‐MEK and this induction was further enhanced by U0126 (Figure 7D). p‐ERK was greatly stimulated by RASSF9, which was almost completely abolished by U0126 (Figure 7D). Accordingly, c‐Myc and Fos were stimulated by RASSF9, and the application of U0126 largely attenuated these stimulations (Figure 7E,F).

**Figure 8:**
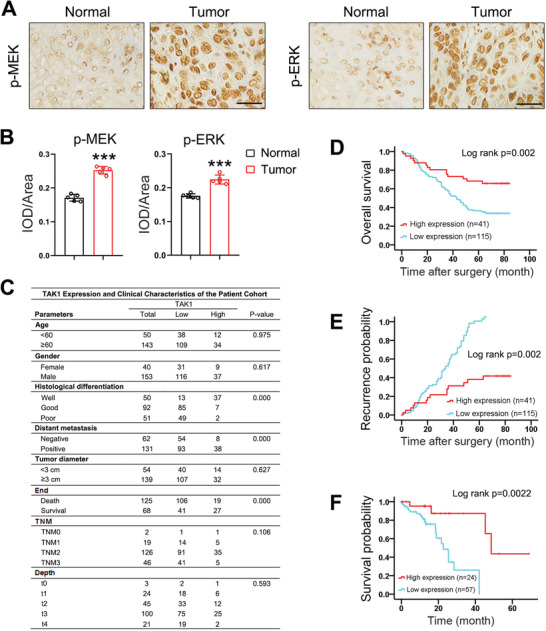
High TAK1 expression in primary esophageal squamous tumor samples is correlated with favorable patient prognosis. A) Expression of p‐MEK and p‐ERK in esophageal squamous tumor tissues and adjacent normal tissues. Representative images were shown (*n* = 5). B) Quantitative analysis of p‐MEK and p‐ERK expression as shown in (A). *n* = 5. IOD: integral optical density. C) Esophageal squamous cancer patient demographics and clinical information. D,E) Esophageal squamous cancer patients with high TAK1 expression have longer survival time (D) and lower probability of recurrence (E) after surgery compared with patients with low TAK1 expression. F) TCGA database showing survival time in ESCC patients is reversely correlated with TAK1 expression. Scale bar = 200 µm. Data are presented as mean ± SD (error bars). Statistical significance was tested by two‐tailed unpaired Student *t*‐test (B) or log‐rank (Kaplan–Meier) test (D–F). ***p < 0.001.

Given that TAK1 expression is reduced in esophageal squamous tumor tissues (Figure 1A–D), RASSF9‐mediated KRAS dimerization would be strengthened, which would then activate the downstream signaling pathway including MEK and ERK. To further confirm this notion, we measured these protein levels in the transplanted tumors (Figure 3). As expected, increased expression of TAK1 induced by LV‐TAK1 stimulated p‐RASSF9, whereas p‐MEK and p‐ERK were reduced (Figure 7G). On the contrary, TAK1 knockdown induced a decrease in p‐RASSF9; meanwhile, p‐MEK and p‐ERK were increased (Figure 7H). In human samples, our immunohistochemical results showed that both p‐MEK and p‐ERK were robustly enhanced in the tumor tissues (**Figure** **8**A,B). Together, these results strongly indicate that cell proliferation induced by RASSF9 is mediated through the activation of the RAS/RAF/MEK/ERK axis.

### 2.6 TAK1 Negatively Correlates with Esophageal Squamous Tumor Patient Survival

Our data thus far showed that TAK1 was a negative regulator of esophageal squamous tumor cell growth and proliferation. We thus hypothesized that TAK1 expression may be closely correlated with the clinical outcomes. A patient cohort with 193 individuals diagnosed with esophageal cancer was employed to validate this hypothesis. Figure 8C shows the key clinical characteristics of the patients in the cohort. Using Kaplan–Meier survival analysis, we found a significant difference (*p* = 0.002, log‐rank test) in 5‐year cancer‐specific survival between patients with low and high expression of TAK1 (Figure 8D). We found that probability of recurrence was lower in patients with high expression of TAK1 compared to those with low expression of TAK1 (Figure 8E). Moreover, human TCGA database also revealed that low expression of TAK1 was correlated with poorer survival in ESCC patients (Figure 8F). These clinical survey data further confirm that TAK1 is a negative regulator of esophageal squamous tumor progression.

## 3 Discussion

TAK1 is an evolutionarily conserved member of the mitogen‐activated protein kinase (MAPK) family. Accumulating evidence supports an association between dysregulated expression of TAK1 and many human diseases including cancer.^[^ ^7^, ^9^, ^11^, ^13^, ^14^, ^15^, ^17^ ^]^ TAK1 was initially discovered as a protein that mediates TGF*β* and bone morphogenetic protein signaling transduction.^[^ ^31^ ^]^ Subsequent studies have shown that TAK1 plays a pivotal role in tumor microenvironment construction and thus affects cancer progression.^[^ ^32^ ^]^ In this study, we compared the expression level of TAK1 between esophageal squamous tumor and normal tissues and found that TAK1 was expressed at lower levels in tumor tissues. Our observation is in agreement with a previous study showing that TAK1 expression was progressively reduced with increasing Gleason grade in 50 well‐characterized human prostate cancer specimens.^[^ ^16^ ^]^ Studies on TAK1 expression in tumor and normal tissues are absent for other types of cancers, such as colon cancer, melanoma, and breast cancer, although the roles of TAK1 in tumorigenesis have been extensively characterized.^[^ ^9^, ^10^, ^11^, ^33^ ^]^ Our clinical data shows that low expression of TAK1 in esophageal squamous cancer patients is strongly correlated with lower survival rate and higher recurrence probability. Human TCGA database also revealed such a correlation between TAK1 expression and survival in ESCC patients. These evidences suggest that TAK1 may be a potential indicator for prognosis and/or diagnosis of ESCC. However, this notion needs further confirmation using larger clinical sample size together with long time follow‐up surveys. On the contrary, the another study showed that elevated expression of TAK1 correlates with reduced disease free survival in patients diagnosed with primary melanoma or colon cancer, indicating that high expression of TAK1 presents a risk factor for disease progression.^[^ ^9^, ^10^ ^]^ We speculate that this discrepancy in results may be due to the different kinds of cancers studied (esophageal squamous cancer vs melanoma).

As a key node integrating diverse signaling pathways, TAK1 plays an important role in cell fate regulation.^[^ ^8^ ^]^ TAK1 is commonly characterized as a tumor suppressor in several types of cancers. For instance, TAK1 ablation in liver parenchymal cells triggered hepatocyte apoptosis in a caspase‐dependent manner.^[^ ^14^ ^]^ In colon cancers, TAK1 is required for tumor cell viability, and inhibition of TAK1 activity promoted tumor cell apoptosis.^[^ ^11^ ^]^ Inhibition of TAK1 with Oxo, expression of inactive TAK1, or deletion of Map3k7 were individually sufficient to sensitize melanoma cells to cell death induced by TNF*α* or TRAIL‐based combination treatment.^[^ ^9^ ^]^ However, we unexpectedly found that blockade of TAK1 using siRNA‐ or CRISPR/Cas9‐mediated gene silencing largely promoted esophageal squamous tumor cell proliferation, whereas elevated expression of TAK1 retarded cell growth. Our observations are consistent with a previous study which showed that TAK1 loss increased cell proliferation, migration, and invasion in both murine prostate stem cells and human prostatic epithelial cells.^[^ ^16^ ^]^ Thus, the discrepancies between our study and other studies^[^ ^9^, ^11^, ^14^ ^]^ likely arise from pleiotropic activities of TAK1 in different cell types. It is worthy to note that, the decreased cell proliferation induced by TAK1 may be arisen from enhanced cell apoptosis. Indeed, our data showed that TAK1 promotes cell apoptosis in ECA‐109 cells, and knockdown of TAK1 reduces cell apoptosis. However, our data from EdU incorporation, cell colony formation and Ki67 expression clearly showed that TAK1 has a negative impact on cell proliferation in esophageal squamous tumor cells.

Recent studies have shown that TAK1 uses MAPKs and nuclear factor *κ*B as two main downstream targets to execute its tumor suppression or activation activities.^[^ ^8^, ^9^, ^10^, ^14^, ^16^, ^34^ ^]^ In this study, we performed co‐immunoprecipitation coupled with mass spectrometry and found RASSF9 to be a downstream target of TAK1. Based on the location of the RA domain, the RASSF family is divided into two groups: C‐terminal RASSFs (RASSF1‐6) and N‐terminal RASSFs (RASSF7‐10).^[^ ^35^ ^]^ Previous studies have shown that C‐RASSFs are downregulated in human cancers and this downregulation is often correlated with tumor progression.^[^ ^18^, ^36^ ^]^ Compared to C‐RASSFs, N‐RASSFs have rarely been examined. Our study showed that RASSF9, a member of N‐RASSFs, is a target of TAK1 and transduces its inhibitory effect on esophageal tumor cell growth. Increased expression of RASSF9 augmented tumor cell proliferation, whereas knockdown of RASSF9 resulted in the opposite outcome. Our results suggest that unlike C‐RASSFs, RASSF9 is an activator of esophageal tumor cell growth. Consistent with our results, RASSF7 was found to localize to the centrosome and knockdown of RASSF7 led to a failure in the formation of mitotic spindle and caused mitotic arrest of cells.^[^ ^24^ ^]^ On the contrary, two recent studies have shown that RASSF9 was reduced in breast and gastric cancers and RASSF9 inhibits these cancer cell proliferation.^[^ ^27^, ^37^ ^]^ The reason for this discrepancy may be due to the different cell types.

RAS GTPases, including HRAS, KRAS, and NRAS, are central hubs for transmitting signals from extracellular stimuli to the interior of the cell.^[^ ^28^ ^]^ Once activated, RAS binds to and activates the RAF family kinases, which in turn initiate downstream signal transduction through a cascade of trans‐phosphorylation of MEK1/2 and ERK1/2.^[^ ^21^, ^28^ ^]^ A growing body of evidence has demonstrated that the RAS‐associated signaling pathway is closely correlated with carcinogenesis by stimulating tumor cell growth, proliferation, and metastasis. It is well documented that the functional form of RAS is a dimer.^[^ ^19^, ^38^ ^]^ The presence of the RA domain in RASSF9 makes the protein a potentially ideal candidate for inducing RAS dimerization and thus activating the RAF/MEK/ERK axis. Consistent with this notion, in this study we showed that elevated expression of RASSF9 induces RAS dimerization, results in a series of trans‐phosphorylation of RAF, MEK, and ERK, and eventually increases the expression levels of c‐Myc and Fos. In the presence of TAK1, these RASSF9‐induced effects were largely absent. We speculate that the inhibitory effect of TAK1 may be due to a conformational change induced by TAK1‐mediated phosphorylation of S284 in RASSF9.

Taken together, in this study, we revealed that TAK1 expression is reduced in esophageal squamous tumor tissues. Knockdown of TAK1 promotes esophageal squamous tumor cell proliferation, while elevated expression of TAK1 inhibits tumor cell growth. We further identified RASSF9 as a downstream target of TAK1 which phosphorylates RASSF9 at S284. Following phosphorylation of RASSF9 by TAK1, RAS dimerization is disrupted, leading to a blockade of RAF/MEK/ERK signal transduction and retardation of tumor cell growth. Taken together, we have revealed a novel role of TAK1 in esophageal squamous tumor cell proliferation and determined that TAK1 mediates this through phosphorylating S284 in RASSF9 to weaken the RAS/RAF/MEK/ERK axis‐related signal transduction in cell growth.

## 4 Experimental Section

##### Human ESCC Specimens

For this study, a cohort of 193 patients with ESCC was consecutively recruited from the Affiliated Hospital of Nantong University (Nantong, China). The patients were between 43 and 82 years of age at the time of initial diagnosis. All diagnoses were pathologically confirmed. None of the patients had received radiotherapy, chemotherapy, or immunotherapy prior to the surgery. Following surgical excision, all the fresh tissues (ESCC and matched adjacent tissues) were immediately washed with sterile phosphate buffered saline (PBS) and immediately fixed in 10% formaldehyde for 12 h prior to being embedded in paraffin or stored at −80 °C for protein and RNA extraction. Complete follow‐up of each patient was available for at least 5 years. During the follow‐up period, diagnosis of distant metastasis was based on imaging methods. Overall survival was defined as the time elapsed from surgery to the death of the patient. Follow‐up information of all participants was updated every 3 months through telephone enquiry and questionnaire letters. Information pertaining to the death of patients was obtained from the family and verified by review of public records. All patients received written informed consent. This study was approved by the Ethics Committee of Affiliated Hospital of Nantong University.

##### Animal Experiments

Male BALB/c nude mice (6 weeks old, weighing 18–22 g) were purchased from Shanghai Slake Laboratory Animal Co. Ltd. (Shanghai, China). The mice were housed five per cage and maintained under specific pathogen free conditions in 12/12 h light and dark cycle. All animal experiments were performed according to the institutional ethical guidelines of animal care, with the approval of the Animal Experimentation Ethics Committee of the Nantong University. For the subcutaneous injection model, 2 × 10^6^ ECA‐109 cells (TAK1 knockdown or overexpression, and negative control) diluted in 100 µL PBS were implanted into the axilla of BALB/c nude mice (*n* = 6 to 10 per group). Tumor growth was measured every 3 days using a slide caliper. The volume of the tumors was calculated using the formula: Tumor volume (TV) = 1/2 length × width × width. All the mice were sacrificed after one month, and the tumor tissues were excised for further measurements. Subsequently, tumor volume and mass were measured to compare the tumor growth rate in each group. All experiments involving animals were approved by the Institutional Animal Care and Use Committees of the Nantong University (Approval ID: SYXK [SU] 2017‐0046).

##### Cell Culture

All human ESCC cell lines including ECA‐109, TE‐1, KYSE‐150, and the normal human esophageal epithelial cell line (HEEC) were obtained from Shanghai Cell Bank (Shanghai Biological Sciences, Chinese Academy of Sciences, Shanghai, China). HEK293 cells were purchased from ATCC (Manassas, VA, USA). All cell lines were authenticated by morphological observation under a microscope and tested using short tandem repeat profiling. The cells were cultured in Dulbecco's modified Eagle's medium supplemented with 10% FBS, 1% penicillin/streptomycin, and incubated at 37 °C in a humidified atmosphere with 5% CO_2_.

##### Plasmid Construction

The coding sequences of Map3k7 and Rassf9 were synthesized by Heyuan Biotechnology Company (Shanghai, China). The synthesized Map3k7 was integrated into pcDNA3.1(+) (Invitrogen, Carlsbad, CA, USA) vector using EcoR I and Xho I. The synthesized Rassf9 was cloned into pcDNA3.1(+) at the sites of Hind III and BamH I. S protein (SP) tag was generated by PCR and incorporated into pcDNA3.1(+)‐RASSF9 at the amino terminus by Hind III and BamH I. SP‐tagged pcDNA3.1(+) was generated by the same method. TAK1 (K63W) and RASSF9 (S284A) were generated using a PCR‐based mutagenesis kit (Stratagene, La Jolla, CA, USA) using pcDNA‐TAK1 and pcDNA‐RASSF9 as templates, respectively. The primer sequences are listed in Table S1, Supporting Information. pcDNA‐CFP‐KRAS4B and pcDNA‐YFP‐KRAS4B were gifts from Kenneth Westover (Addgene plasmid #112 717 and #112 718).^[^ ^19^ ^]^ All plasmids were confirmed by sequencing.

##### Generation of Antibodies against RASSF9 and Phospho‐RASSF9 (S284)

Rabbit polyclonal antibodies that recognize RASSF9 and anti‐phospho‐RASSF9 (S284) were raised against DKLSAEIEKEVKSVC and DKL(pS)AEIEKEVKSVC peptides, respectively at GenScript (Piscataway, NJ, USA).

##### In Vitro Kinase Assay

Recombinant RASSF9 (2 µg) was incubated with 1 µg of recombinant TAK1 (Novus, H00006885‐P01) in kinase assay buffer (Cell Signaling, #9802) containing 25 mm Tris‐HCl (pH 7.5), 5 mm beta‐glycerophosphate, 2 mm dithiothreitol, 0.1 mm Na_3_VO_4_, 10 mm MgCl_2_, and 200 µm ATP (Cell Signaling, #9804). The incubation was performed for 30 min at 37 °C. The reaction was terminated by adding 1 × Laemmli buffer. The samples were boiled at 100 °C for 5 min and separated by sodium dodecyl sulfate polyacrylamide gel electrophoresis (SDS‐PAGE), and then subjected to Coomassie blue staining or transferred onto a polyvinylidene fluoride membrane. Signals for RASSF9, p‐RASSF9, and TAK1 were detected using the respective antibodies.

##### CRISPR‐Cas9‐Knockout

To evaluate the effect of Map3k7 knockout in ECA‐109 cells, an episomal vector‐based CRISPR/Cas9 system was established as previously described.^[^ ^39^ ^]^ We designed an all‐in‐one OriP/EBNA1‐based vector (epiCRISPR), that expressed a gRNA, Cas9, and a puromycin resistance gene (for enrichment of the transfected cells through drug selection). ECA‐109 cells were transfected with the plasmids using Lipofectamine 2000 transfection reagent (Invitrogen) according to the manufacturer's instructions. Cells were selected using 2.5 µg mL^−1^ puromycin for 1 week. The surviving stable cells were used for further experiments. The gRNA sequence is listed in Table S1, Supporting Information.

##### FRET Assay

Briefly, ECA‐109 cells were plated in 4‐well chambered coverglass at a density of 2 × 10^4^ cells/well and transfected with YFP‐KRAS, CFP‐KRAS, RASSF9, RASSF9 S284A, and TAK1 as indicated. 24 h post‐transfection, the cells were subjected to serum starvation for 22 h, followed by treatment with EGF (10 ng mL^−1^) for 30 min, and then visualized under a microscope. Live cell imaging was performed using a Confocal/Multiphoton microscope (Zeiss, LSM880). CFP was excited with 405 nm light and emission was monitored over 460–492 nm range; YFP was excited with 514 nm light and emission was monitored over 526–589 nm range. YFP was photobleached using a 514 nm laser line at 80% power for 1 min. An image of the CFP and YFP fluorescence after photobleaching was obtained using the respective filter sets. Data were collected from 10 to 12 different cells from different fields from the same well of the chambered coverglass. Two to three regions of interest in the photobleached area per cell were selected for CFP fluorescence measurements before and after photobleaching. Quantitation was performed using ZEN software (ZEISS).

##### Immunohistochemical Quantitation

The digitized images of immunohistochemistry were quantitatively analyzed using Image‐Pro Plus 6.0 (IPP 6.0, Media Cybernetics, Rockville, MD, USA). Area represents the total area of the dyed region; Integral optical density (IOD) represents the total optical density of brown‐stained areas; IOD/area represents the expression quantitation of target protein. The signal density of the tissue areas from five randomly selected fields were counted in a blinded manner and subjected to statistical analysis.

##### Statistical Analysis

The in vitro experiments were repeated at least three times. All experimental data are presented as mean ± S.D. The statistical significance of differences was analyzed with unpaired two‐sided Student *t*‐test or one‐way analysis of variance (ANOVA) with Bonferroni correction for multiple group comparisons. Survival analysis was performed using Kaplan–Meier method and log‐rank test. Cox proportional hazards regression model was established to assess the factors independently associated with patient's survival. Statistical analysis was conducted using GraphPad Prism version 8.0 (GraphPad Software, La Jolla, CA, USA) or SPSS 23.0 (SPSS Inc., Chicago, IL, USA). Sample size (*n*) was given in each figure legend. In all cases, *p < 0.05, **p < 0.01, and ***p < 0.001.

More detailed methods are provided in the Supporting Information.

## Conflict of Interest

The authors declare no conflict of interest.

## Author Contributions

H.S. and Q.J. contributed equally to this work. C.S. conceived, designed, and supervised the study. H.S., Q.J., Y.M., Y.W., J.D., and X.L. conducted the experiments. H.S., Q.J., X.T., and C.S. analyzed and interpreted the data. X.T. and C.S. wrote the manuscript with the input from all other authors.

## Supporting information

Supporting Information

Supporting Information (R20180400495_IP_PR‐Phosphorylation)
