# Supplementary material for: TAK1 Phosphorylates RASSF9 and Inhibits Esophageal Squamous Tumor Cell Proliferation by Targeting the RAS/MEK/ERK Axis
Source: Adv Sci (Weinh). 2021 Jan 6;8(5):2001575. doi: 10.1002/advs.202001575 (PMC7927628; doi:10.1002/advs.202001575)
Supplement: Supplementary file 1 — Supporting Information [file ADVS-8-2001575-s001.pdf]

© 2020 Wiley-VCH GmbH

ADVANCED SCIENCE
Open Access

Supporting Information

for *Adv. Sci.,* DOI: 10.1002/advs.202001575

TAK1 Phosphorylates RASSF9 and Inhibits Esophageal Squamous Tumor Cell Proliferation by Targeting the RAS/MEK/ERK Axis

Hui Shi[1], *Qianqian Ju*[1,2], *Yinting Mao*[1,2], *Yuejun Wang*[2], *Jie Ding*[2], *Xiaoyu Liu*[2], *Xin Tang*[2,*], *Cheng Sun1,*[2,*]

Supporting Information

TAK1 phosphorylates RASSF9 and inhibits esophageal squamous tumor cell proliferation by targeting the RAS/MEK/ERK axis

Hui Shi[1], Qianqian Ju[1,2], Yinting Mao[1,2], Yuejun Wang[2], Jie Ding[2], Xiaoyu Liu[2], Xin Tang[2,*], Cheng Sun[1,2,*]

[1] Department of Thoracic Surgery, Nantong Key Laboratory of Translational Medicine in Cardiothoracic Diseases, Institute of Translational Medicine in Cardiothoracic Diseases, Affiliated Hospital of Nantong University, Nantong, China

[2] Key Laboratory for Neuroregeneration of Jiangsu Province and Ministry of Education, Nantong University, Nantong, China.

* Correspondence: suncheng1975@ntu.edu.cn (C.S.) or tangxin@ntu.edu.cn (X.T.)

Hui Shi and Qianqian Ju have contributed equally to this work.

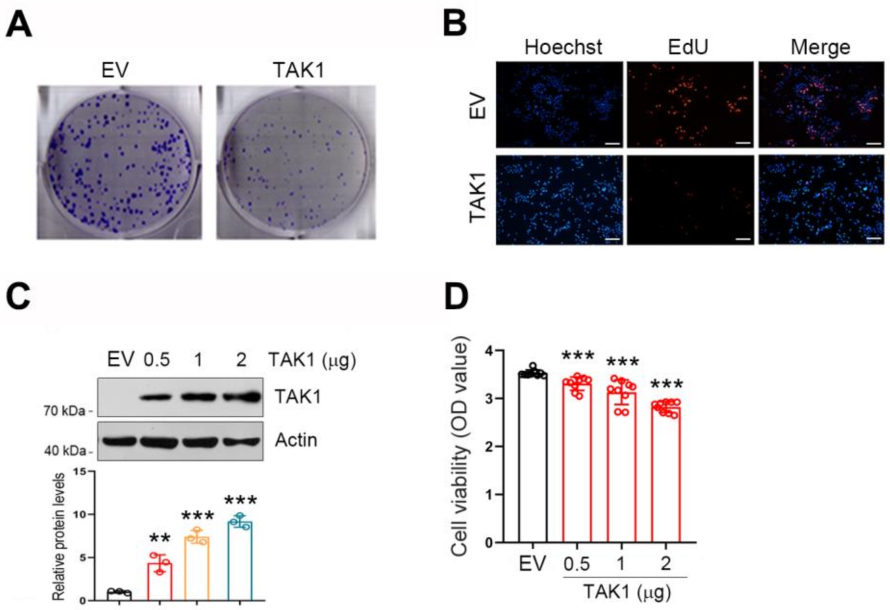

Figure S1. TAK1 negatively regulates cell proliferation in ECA-109 cells. A) Elevated TAK1 expression blocks cell colony formation. Representative images were shown (n = 4 biologically independent replicates per group). B) Elevated TAK1 expression blocks EdU incorporation. Representative images were shown (n = 5 biologically independent replicates per group). Scale bar = 500 μm. C) TAK1 increases in a dose-dependent manner in cells transfected with plasmid expressing TAK1. Protein levels were analyzed by western blot and Actin was used as a loading control. Representative blots were shown (n = 3 biologically independent replicates per group). D) Cell viability was reduced by TAK1 in a dose-dependent manner (n = 9 biologically independent replicates per group). EV: empty vector. Data are presented as means ± SD (error bars). Statistical significance was analyzed by two-tailed unpaired Student *t*-test. ** P < 0.01, *** P < 0.001 vs EV.

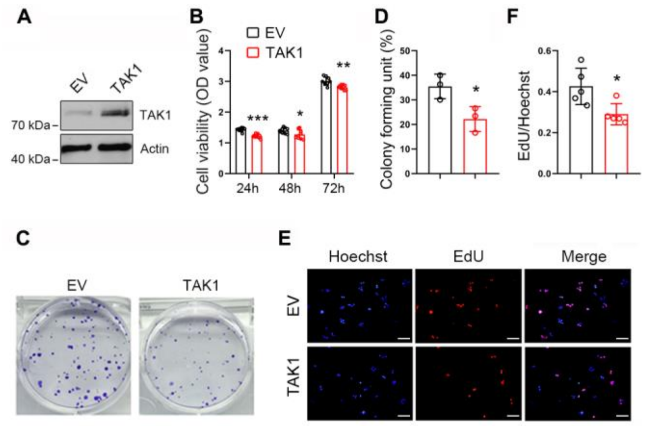

Figure S2. Elevated expression of TAK1 reduces cell proliferation in TE-1 cells. A) TE-1 cells were transfected with plasmid expressing TAK1. Western blot analysis showing TAK1 was increased by the transfection. Actin was used as a loading control. Representative blots were shown (*n* = 3 biologically independent replicates per group). B) Enhanced TAK1 decreases cell viability (*n* = 9 biologically independent replicates per group). C-F) Elevated TAK1 expression blocks cell colony formation (C-D; *n* = 3 biologically independent replicates per group) and EdU incorporation (E-F; *n* = 5 biologically independent replicates per group). Representative images were shown. Scale bar = 500 μm. EV: empty vector. Data are presented as means ± SD (error bars). Statistical significance was analyzed by two-tailed unpaired Student *t*-test. * P < 0.05, ** P < 0.01 and *** P < 0.001 vs EV.

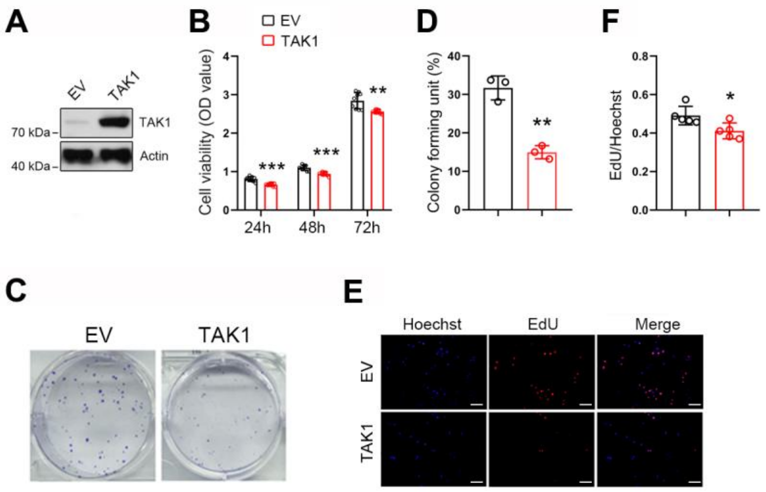

Figure S3. Elevated expression of TAK1 reduces cell proliferation in KYSE-150 cells. A) KYSE-150 cells were transfected with plasmid expressing TAK1. Western blot analysis

showing TAK1 was increased by the transfection. Actin was used as a loading control. Representative blots were shown (*n* = 3 biologically independent replicates per group). B) Enhanced TAK1 decreases cell viability (*n* = 9 biologically independent replicates per group). C-F) Elevated TAK1 expression blocks cell colony formation (C-D; *n* = 3 biologically independent replicates per group) and EdU incorporation (E-F; *n* = 5 biologically independent replicates per group). Representative images were shown. Scale bar = 500 μm. EV: empty vector. Data are presented as means ± SD (error bars). Statistical significance was analyzed by two-tailed unpaired Student *t*-test. * P < 0.05, ** P < 0.01 and *** P < 0.001 vs EV.

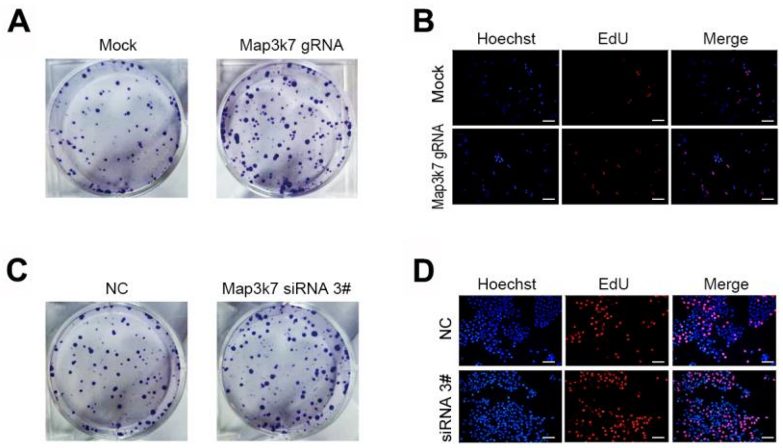

Figure S4. Decreased expression of TAK1 stimulates cell proliferation in ECA-109 cells. A-B) CRISPR-Cas9 induced TAK1 deficiency promotes cell colony formation (A) and EdU incorporation (B). C-D) siRNA-mediated knockdown of TAK1 enhances cell colony formation (C) and EdU incorporation (D) in ECA-109 cells. Representative images were shown (*n* = 4 biologically independent replicates per group in A, C; *n* = 5 biologically independent replicates per group in B, D). Scale bar = 500 μm.

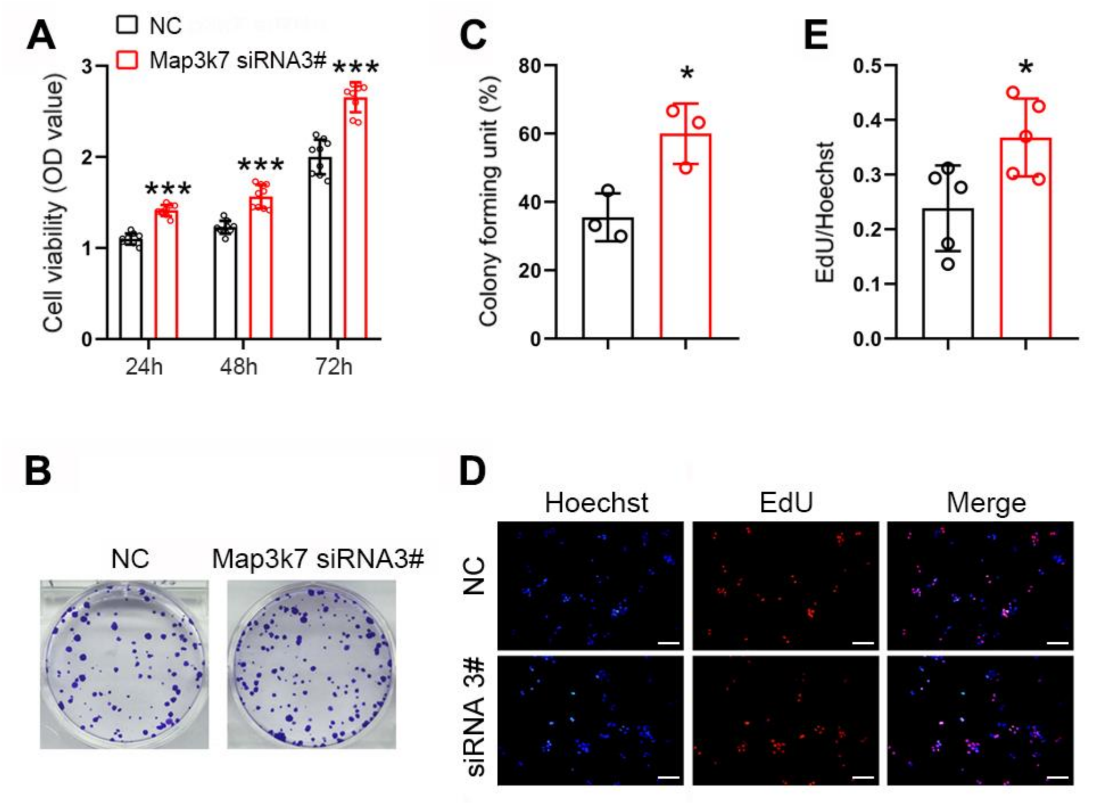

Figure S5. Decreased expression of TAK1 stimulates cell proliferation in TE-1 cells. A) TAK1 knockdown promotes cell viability (*n* = 9 biologically independent replicates pre group). B-C) siRNA-mediated knockdown of TAK1 increases cell colony formation (*n* = 3 biologically independent replicates per group). D-E) siRNA-mediated knockdown of TAK1 boosts EdU incorporation (*n* = 5 biologically independent replicates per group). Representative images were shown. Scale bar = 500 μm. NC: negative control. Data are presented as means ± SD (error bars). Statistical significance was analyzed by two-tailed unpaired Student *t*-test. * P < 0.05, *** P < 0.001 vs NC.

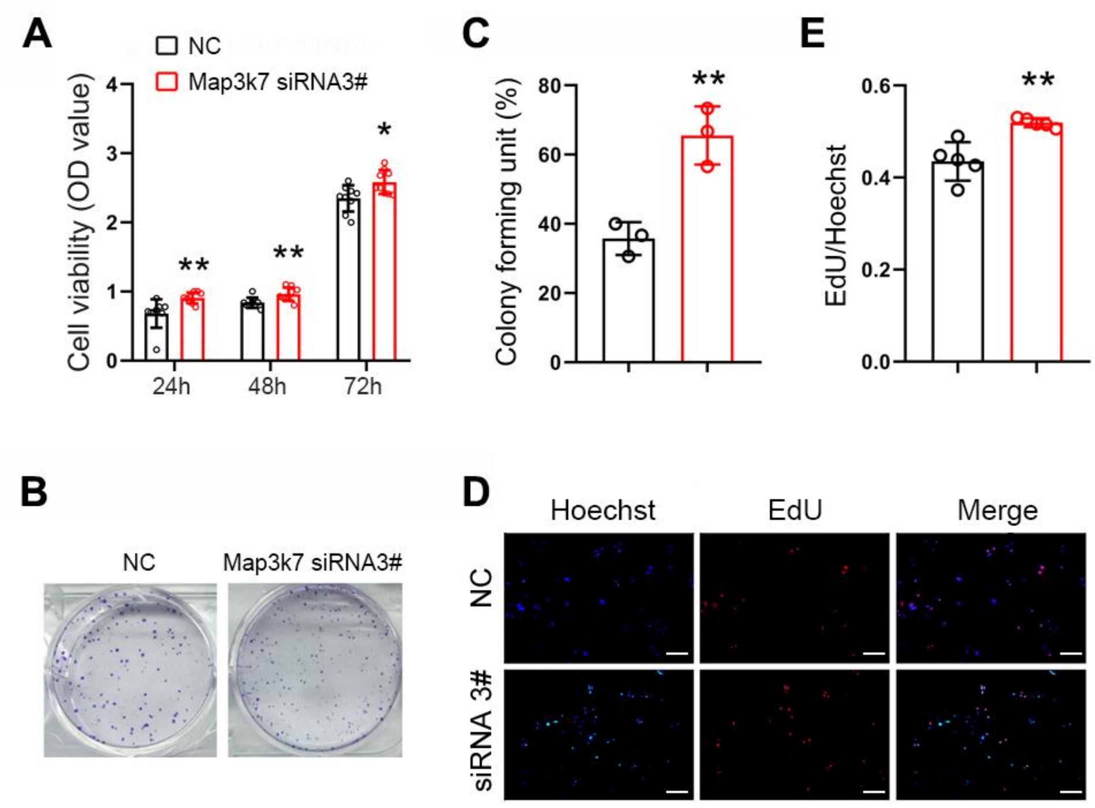

Figure S6. Decreased expression of TAK1 stimulates cell proliferation in KYSE-150 cells. A) TAK1 knockdown increases cell viability (n = 9 biologically independent replicates pre group). B-C) Cell colony formation was enhanced by siRNA-mediated knockdown of TAK1 (n = 3 biologically independent replicates pre group). D-E) Knockdown of TAK1 stimulates EdU incorporation (n = 5 biologically independent replicates pre group). Representative images were shown. Scale bar = 500 μm. NC: negative control. Data are presented as means ± SD (error bars). Statistical significance was analyzed by two-tailed unpaired Student t-test. * P < 0.05, ** P < 0.01 vs NC.

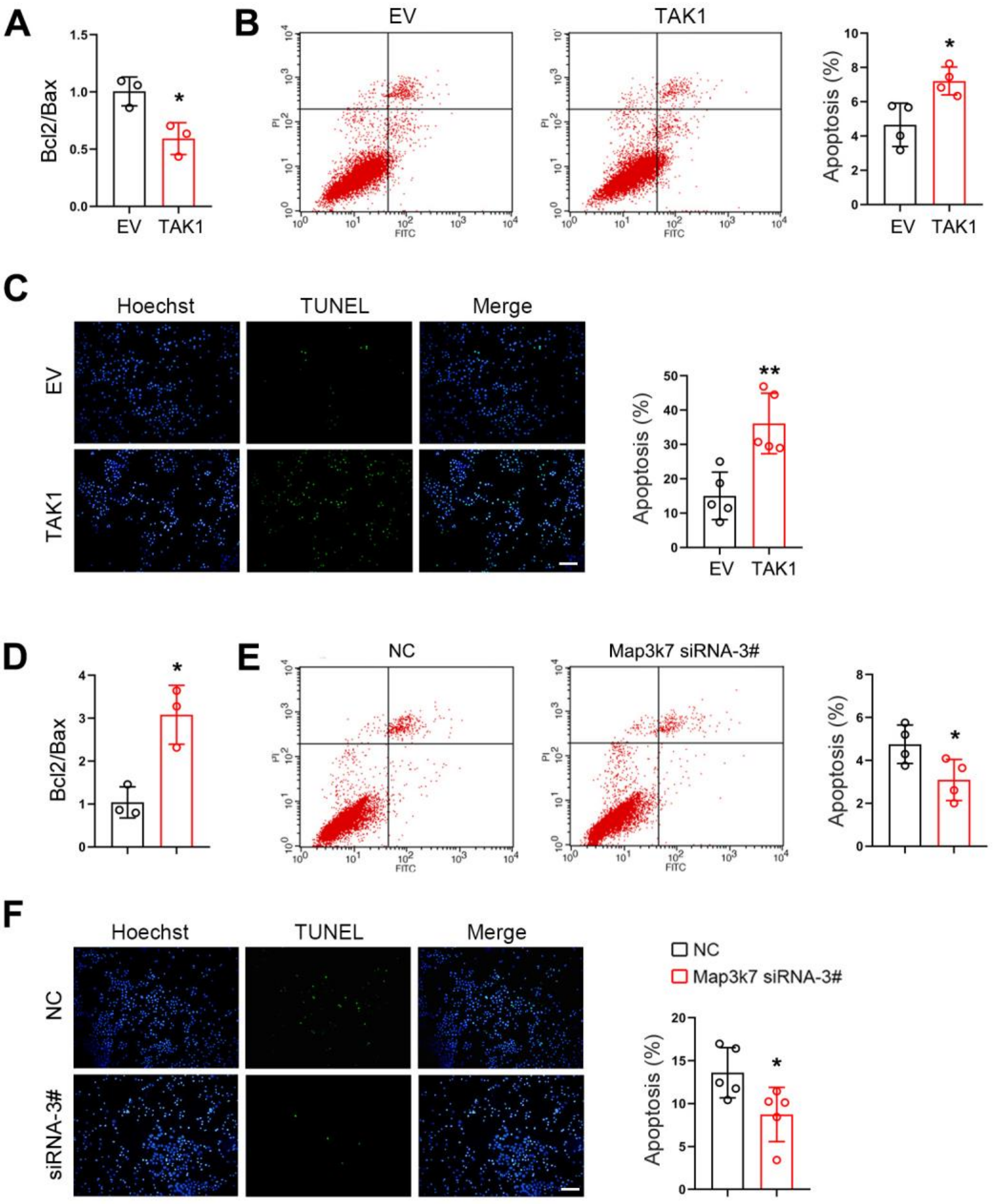

Figure S7. TAK1 stimulates cell apoptosis in ECA-109 cells. A-C) ECA-109 cells were transfected with a plasmid expressing TAK1. 36 h post-transfection, cells were subjected to cell apoptosis analysis by Bcl2/Bax (A; *n* = 3 biologically independent replicates per group), flow cytometry (B; *n* = 4 biologically independent replicates per group) and TUNEL staining (C; *n* = 5 biologically independent replicates per group). D-F) ECA-109 cells were tranfected with Map3k7 siRNA-3# for 72 h, and then cell apoptosis was analyzed by Bcl2/Bax (D; *n* = 3 biologically independent replicates per group), flow cytometry (E; *n* = 4 biologically independent replicates per group) and TUNEL staining (F; *n* = 5 biologically independent replicates per group). Representative images were shown. Scale bar = 500 μm. The mRNA

levels of Bcl2 and Bax were analyzed by qRT-PCR. EV: empty vectior. NC: negative control. Data are presented as means ± SD (error bars). Statistical significance was analyzed by two-tailed unpaired Student *t*-test. * P < 0.05, ** P < 0.01.

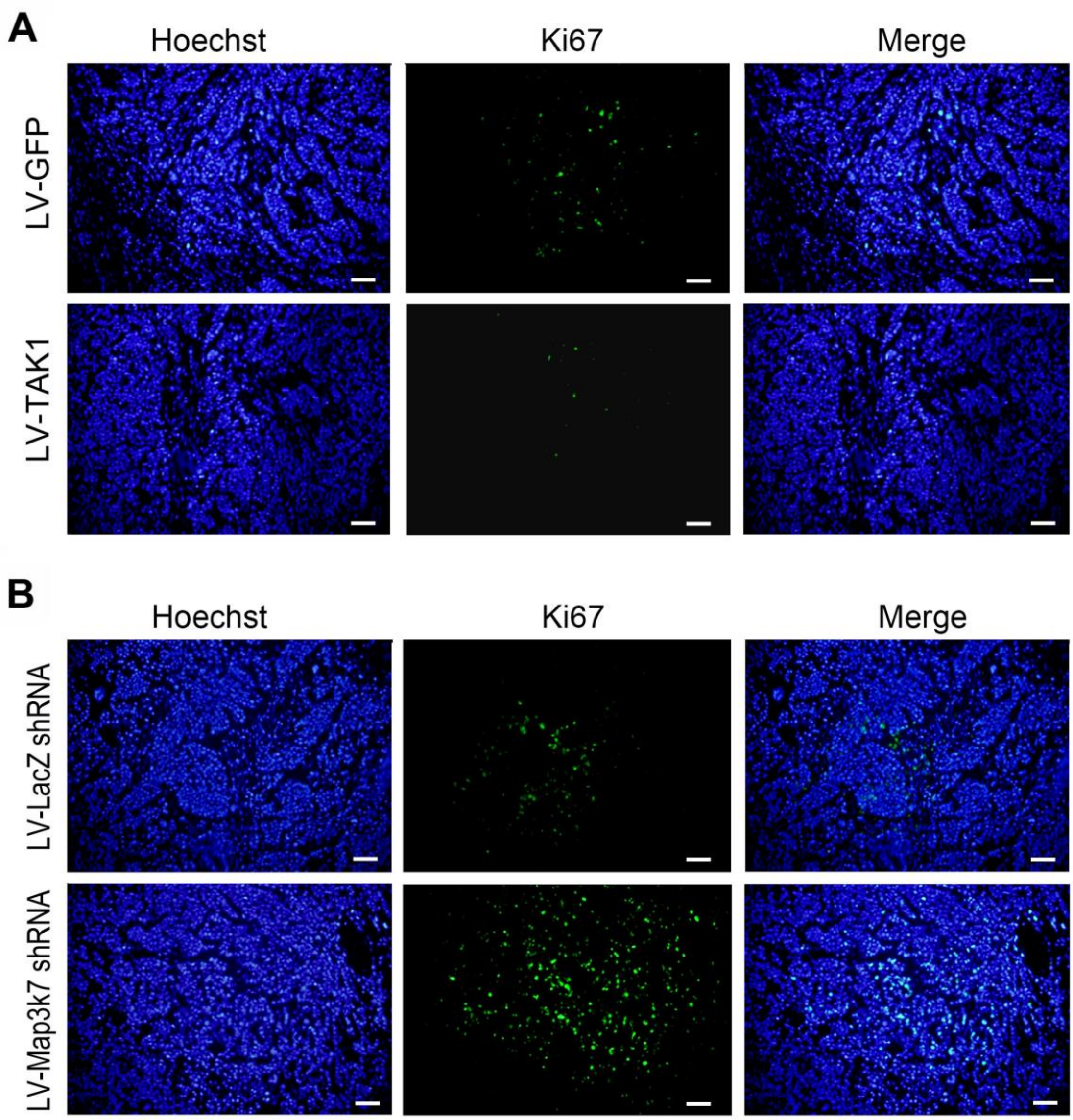

Figure S8. Immunostaining of Ki67 in transplanted tumors. A) Immunostaining of Ki67 in the tumors transduced with LV-TAK1. B) Immunostaining of Ki67 in the tumors transduced with LV-Map3k7 shRNA. Hoechst was used to stain DNA in nucleus. Representative images were shown (*n* = 5). Scale bar = 500 μm.

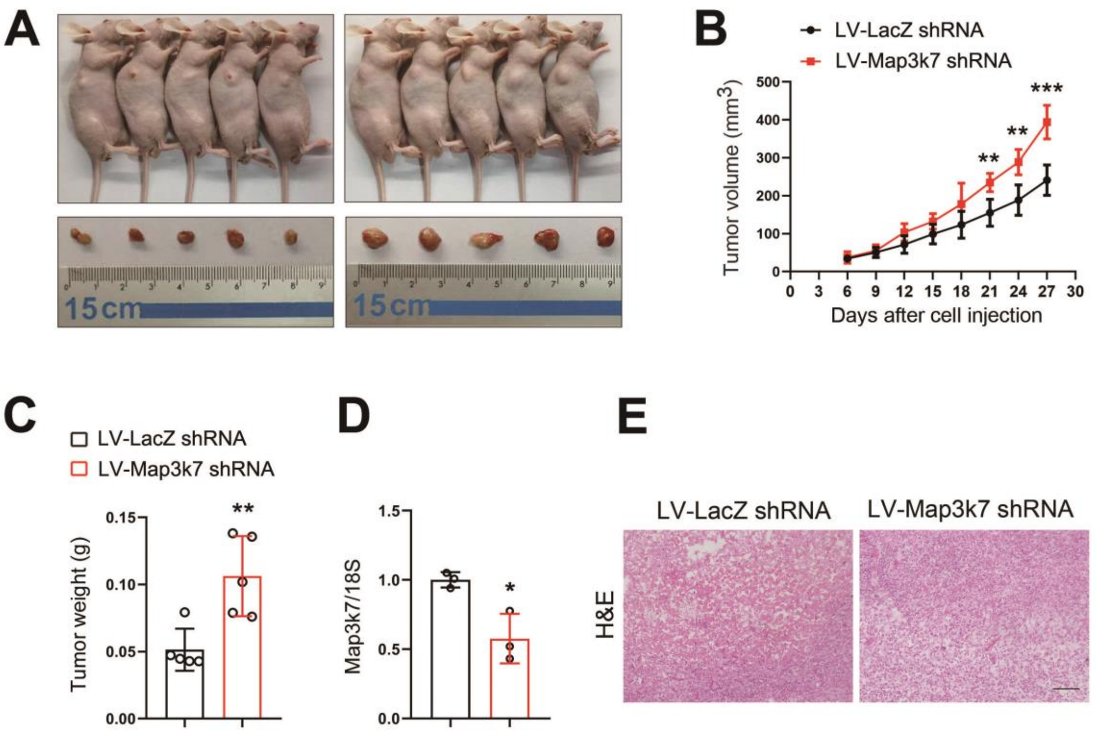

Figure S9. TAK1 knockdown potentiates tumor growth *in vivo*. TE-1 cells were transduced with LV-*Map3k7* shRNA. After selection using puromycin, the cells were transplanted into nude mice. A) Morphology of the nude mice transplanted with tumor cells and explanted tumors. *n* = 5. B-C) TAK1 knockdown increases tumor volume (B) and tumor weight (C). *n* = 5. D) The mRAN levels of TAK1 were decreased by LV-*Map3k7* shRNA. Gene expression was analyzed by qRT-PCR and 18S was used as an internal control. *n* = 3. E) H&E staining. Representative images were shown (*n* = 2). Scale bar = 500 μm. Data are presented as means ± SD (error bars). Statistical significance was analyzed by two-tailed unpaired Student *t*-test. * P < 0.05, ** P < 0.01, *** P < 0.001.

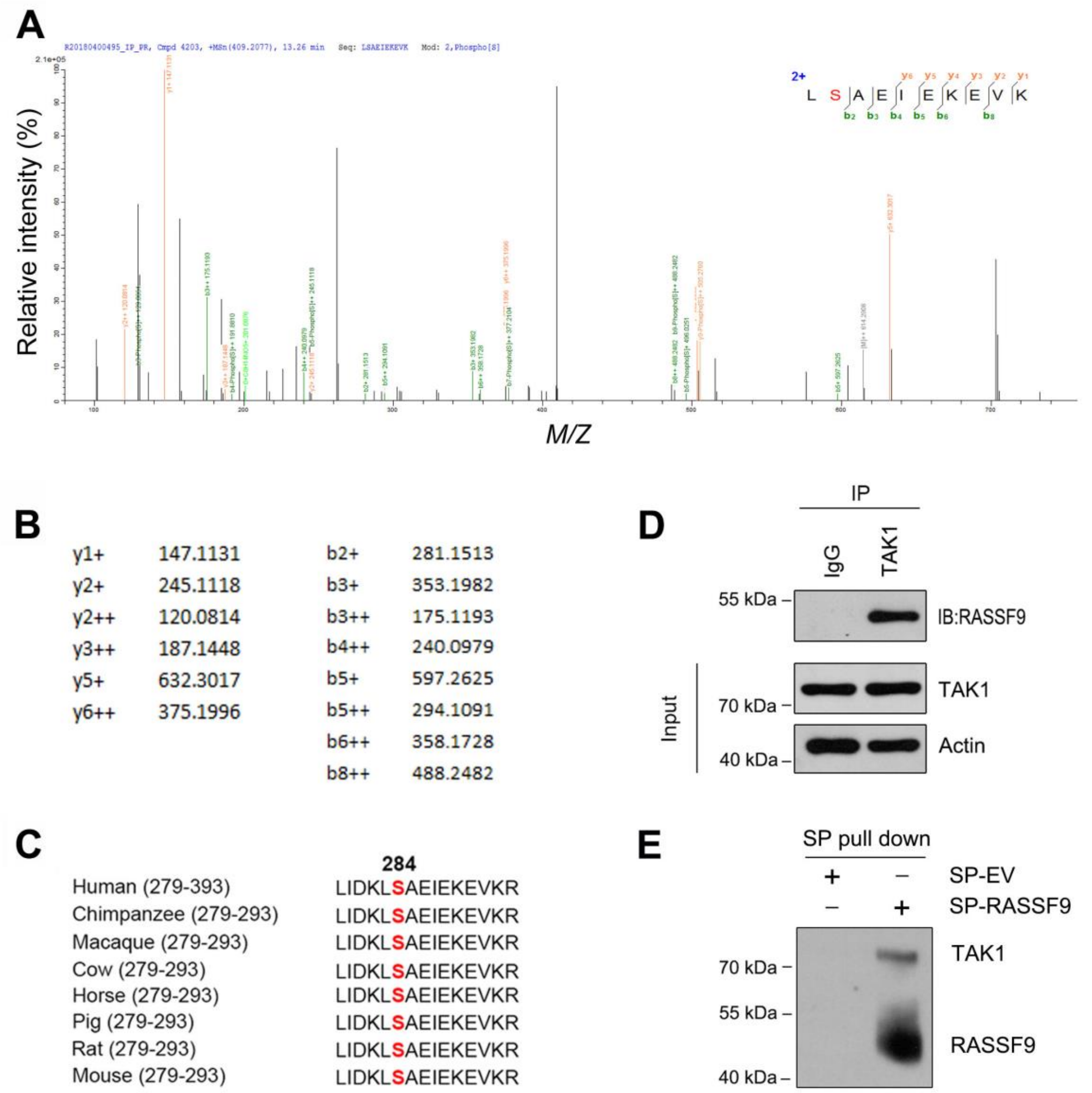

Figure S10. Identification of phosphorylation site in RASSF9 by TAK1. A) Tandem mass spectrometry showing Ser284 in RASSF9 was phosphorylated by TAK1 in ECA-109 cells. B) Mass-to-charge ratio (m/z) of the fragments as shown in (A). C) Sequence alignment of the putative phosphorylation site of S284 in RASSF9 from different species. D) Co-immunoprecipitation analysis showing TAK1 interacts with RASSF9. HEK293 cells were transfected with the plasmid expressing TAK1. 24 h post-transfection, cells were harvested for co-immunoprecipitation assay by using an anti-TAK1 antibody. The immuno-complex was subjected to western blot analysis by using an anti-RASSF9 antibody. Meanwhile, the total cell lysates were analyzed by western blot using anti-TAK1 and anti-Actin antibodies. Actin was used as a loading control. E) S protein (SP) pull-down

assay showing RASSF9 interacts with TAK1. HEK293 cells were transfected with SP tagged RASSF9 or empty vector (EV). 24 h post-transfection, cell lysates were prepared and subjected to SP pull-down assay by using S-protein agarose. The eluted samples were analyzed by western blot and TAK1 and RASSF9 were detected simultaneously by using their respective antibody.

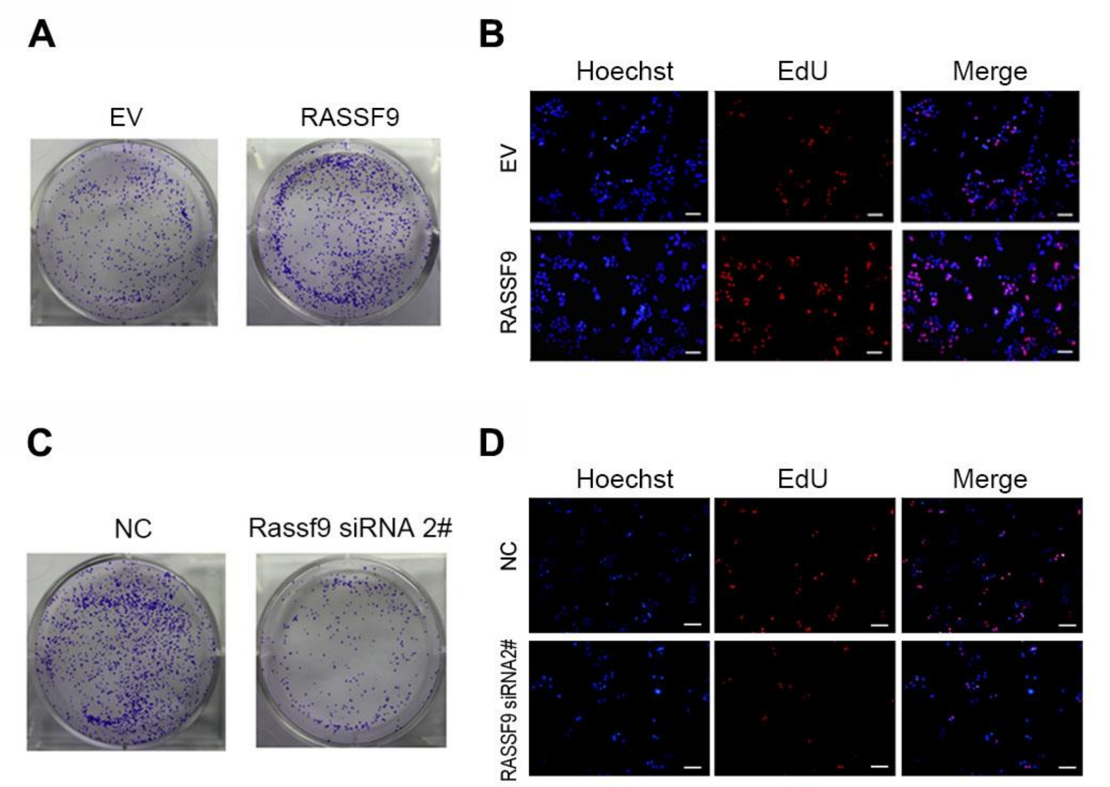

Figure S11. RASSF9 stimulates cell proliferation in ECA-109 cells. A-B) Elevated expression of RASSF9 stimulates colony formation (A) and EdU incorporation (B) in ECA-109 cells. C-D) Knockdown of RASSF9 decreases colony formation (C) and EdU incorporation (D) in ECA-109 cells. EV: empty vector. NC: negative control. Representative images were shown (*n* = 4 biologically independent replicates per group in A, C; *n* = 5 biologically independent replicates per group in B, D). Scale bar = 500 μm.

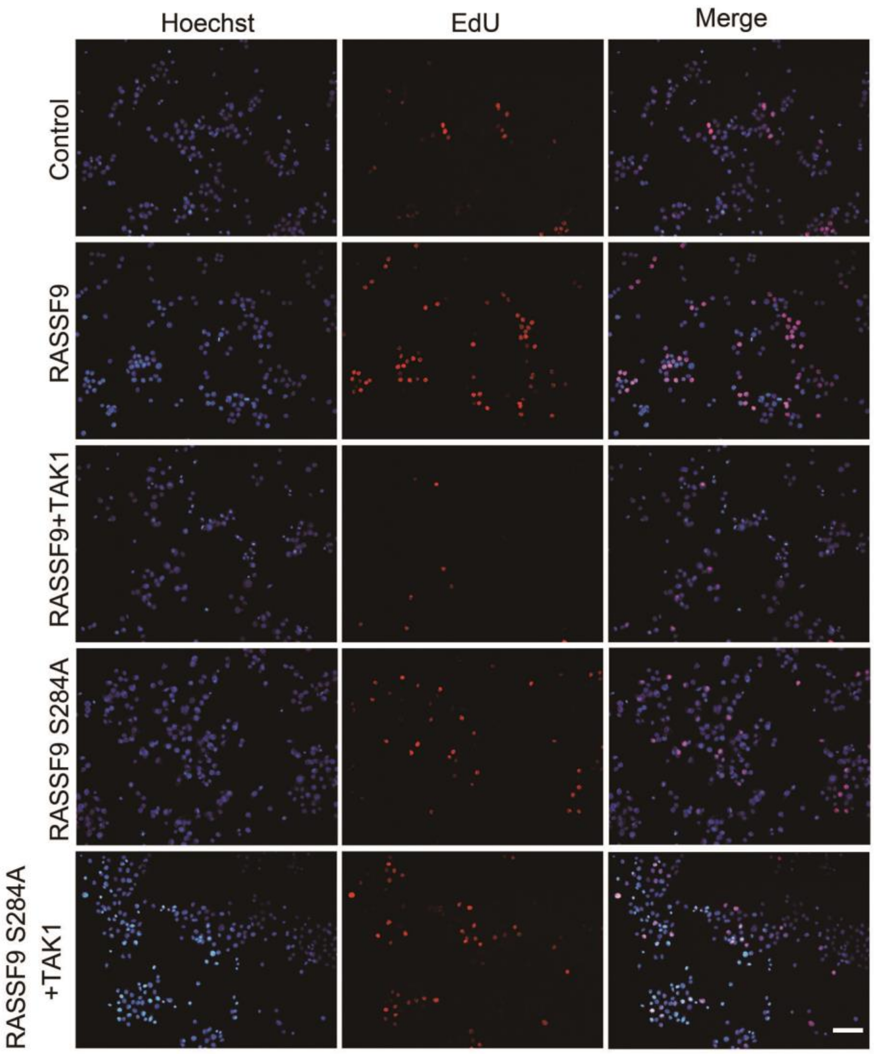

Figure S12. TAK1 inhibition rescues reduced cell proliferation induced by TAK1. ECA-109 cells were transfected with plasmids expressing RASSF9, RASSF9 S284A or TAK1 as indicated. 36 h post-transfection, cell proliferation was assayed by EdU incorporation. Representative images were shown (*n* = 5 biologically independent replicates per group). Scale bar = 500 μm.

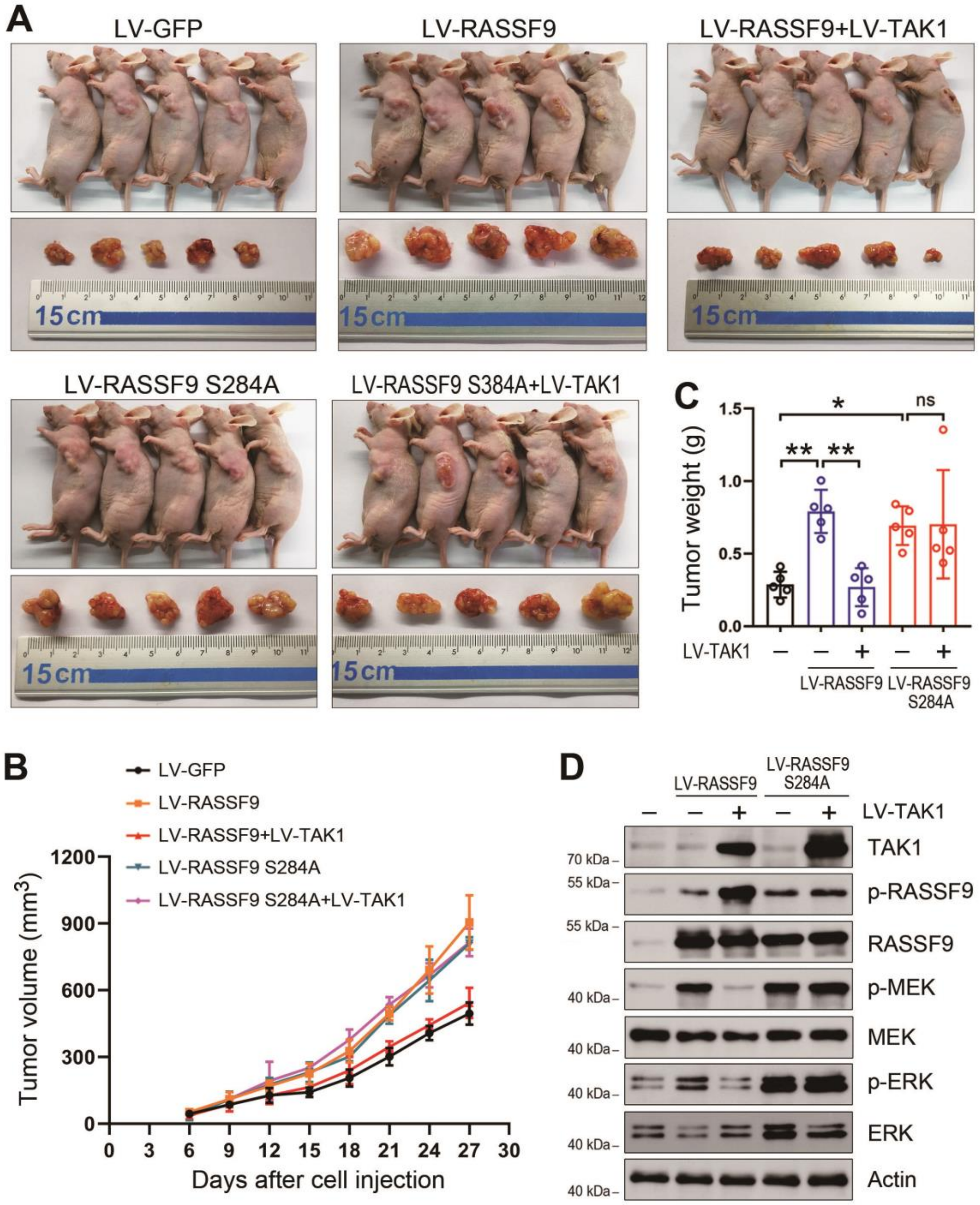

Figure S13. TAK1 has no effect on RASSF9 S284A induced tumor cell growth *in vivo*. ECA-109 cells were transduced with LV-GFP, LV-RASSF9, LV-RASSF9+LV-TAK1, LV-RASSF9 S284A, or LV-RASSF9 S284A+LV-TAK1. After selection using puromycin, the cells were transplanted into nude mice. A) Morphology of the nude mice transplanted with tumor cells and explanted tumors. n = 5. B) Tumor volume. n = 5. C) Tumor weight. n = 5. D) The protein levels of TAK1, p-RASSF9, RASSF9, p-MEK, MEK, p-ERK, ERK in excised tumors were analyzed by western blot. Actin was used as a loading control. Representative blots were shown (n = 2

biologically independent replicates). Data are presented as means ± SD (error bars). Statistical significance was analyzed by two-tailed one-way ANOVA test. * P < 0.05, ** P < 0.01. ns means no significance.

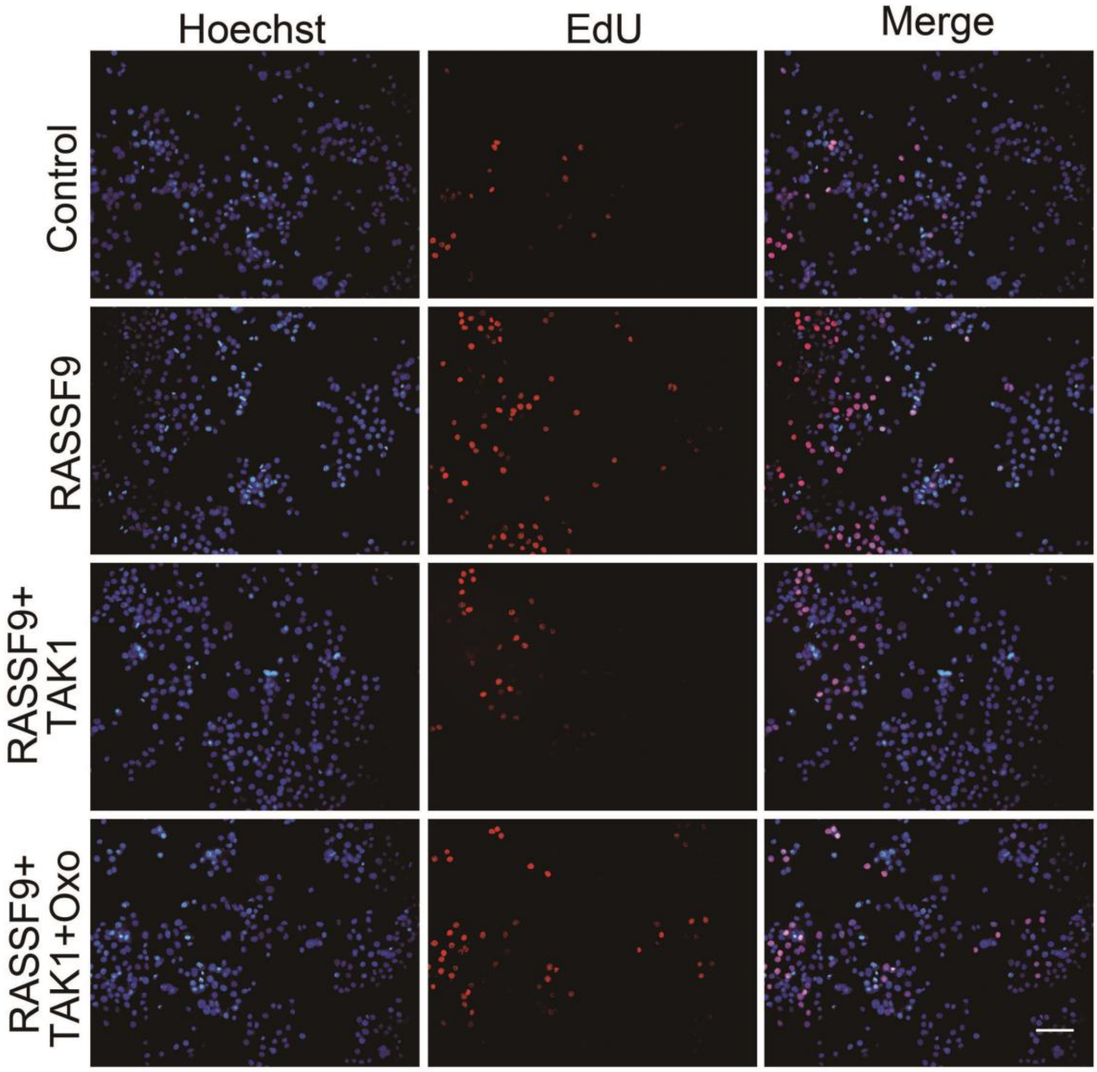

Figure S14. TAK1 inhibition rescues reduced cell proliferation induced by TAK1. ECA-109 cells were transfected with plasmids expressing RASSF9 or TAK1 as indicated. 12 h post-transfection, the cells were treated with 10 μM Oxo for additional 24 h. Representative images were shown (*n* = 5 biologically independent replicates per group). Cell proliferation was assayed by EdU incorporation. Scale bar = 500 μm.

Primer	Sequence (5'—3')
Map3k7	F: ATTGTAGAGCTTCGGCAGTTATC
	R: CTGTAAACACCAACTCATTGCG
Rassf9	F: GAGGACCTGAGCGAAAGTGAT
	R:TCTGGATGCCACTCAAATGAGA
Fos	F: GATACACTCCAAGCGGAGAC
	R: CCCAGTCAGATCAAGGGAAG

Table S1. Primers used in this study.

c-Myc	F: CGACTCTGAGGAGGAACAAG
	R: CGTAGTTGTGCTGATGTGTG
18S rRNA	F: AGTCCCTGCCCTTTGTACACA
	R: CGTTCCGAGGGCCTCACT
Map3k7 siRNA-1	GACTCACTTGATGCGGTAC
Map3k7 siRNA-2	GAGTGAATCTGGACGTTTA
Map3k7 siRNA-3	GGAGTTGTTTGCAAAGCTA
Rassf9 siRNA-1	GGCTACGTTTGGAGAGAAA
Rassf9 siRNA-2	GAAGCCAAATTAGTGCAAA
Rassf9 siRNA-3	GACTCATTGCTTCAGATGA
Map3k7 gRNA	F: ccgAGGGGCTTCGATCATCTCAC
	R: aacGTGAGATGATCGAAGCCCCT
Map3k7 shRNA	Top:
	CACCGGAGTTGTTTGCAAAGCTACGAATAGCTTTGCAAACAACTCC
	Bottom:
	AAAAGGAGTTGTTTGCAAAGCTATTCGTAGCTTTGCAAACAACTCC
TAK1 (K63W)	F:GATGTCGCTATTAGGCAGATAGAAAGTGAGTCTGAGAGGAAGGC
	R:TCAGACTCACTTTCTATCTGCCTAATAGCGACATCTTTTGCTCTCC
RASSF9 (S284A)	F:GACAAGCTGGCCGCCGAGATCGAGAAAGAGG
	R:TCTCGGCGGCCAGCTTGTCGATCAGGATTC

Supporting Methods

Antibodies, Inhibitors, and Growth Factors: The following antibodies were used: rabbit monoclonal antibodies against phospho-MEK1 (Ser298) (EPR3338, ab96379, Abcam, Cambridge, UK), Ki67 (ab15580, Abcam, Cambridge, UK, for IF). Rabbit monoclonal antibodies against TAK1 (D94D7, 5206), phospho-TAK1 (Ser412, 9339), phospho-p44/42 MAPK (Erk1/2) (Thr202/Tyr204, D13.14.4E, 4370), p44/42 MAPK (Erk1/2) (9102), phospho-MEK1/2 (Ser217/221) (41G9, 9154), phospho-c-RAF (Ser338) (56A6, 9427), c-RAF (9422), c-Fos (9F6, 2250), c-Myc (E5Q6W, 18583) and normal rabbit IgG (2729) all from Cell Signaling Technology (Beverley, MA, USA). Mouse monoclonal antibodies against MEK1/2 (L38C12, 4694), β-Actin (8H10D10, 3700) was purchased from Cell Signaling Technology (Beverley, MA, USA). TAK1 inhibitors 5Z-7-oxozeaenol (O9890), NG25 (SML1332) and takinib (SML2216) and an ERK inhibitor U0126 (662009) were purchased from Sigma-Aldrich (St. Louis, MO, USA). A MEK inhibitor Selumetinib (HY-50706) was obtained from MedChemExpress (New Jersey, USA). EGF Recombinant Human Protein (#PHG0311) was purchased from Gibco (Carlsbad, CA, USA).

Recombinant RASSF9: Recombinant human RASSF9 was produced by GenScript (Piscataway, NJ, USA). Briefly, target DNA sequence of RASSF9 was optimized and synthesized. The synthesized sequence was cloned into vector PET-30a(+) with His tag for protein expression in *E. coli*. For producing RASSF9, *E. coli* strain BL21(DE3) was transformed with recombinant plasmid. A single colony was inoculated into TB medium containing related antibiotic; culture was incubated in 37°C at 200 rpm and then induced with IPTG at 15°C for 16 h. Cells were harvested by centrifugation. Cell pellets were resuspended with lysis buffer followed by sonication. The precipitate after centrifugation was dissolved using denaturing agent. Target protein was obtained by one-step purification using Ni column. Target protein was sterilized by 0.22 μm filter before stored in aliquots. The concentration was determined by Bradford protein assay with BSA as standard. The protein purity and molecular weight were determined by standard SDS-PAGE along with western blot confirmation.

Lentivirus Transduction: LV-*Map3k7* shRNA, LV-TAK1, LV-RASSF9, LV-RASSF9 S284A, and LV-GFP were produced at Hanbio Company (Shanghai, China). ECA-109 cells were transduced with these viruses at the dosage of MOI = 10 according to the manufacturer's protocol. Meanwhile, we added 5 μg/ml polybrene in order to improve the transduction efficiency. Transfected ECA-109 cells were selected with 2.5 μg/ml puromycin for 1 week. The efficiency of lentivirus with knockdown or overexpression of TAK1 or RASSF9 were verified by qRT-PCR and western blot analysis.

Quantitative Real Time-PCR (qRT-PCR): Total RNA of cells or tumor tissues were extracted using TRI Reagent according to the manufacturer's instructions, then quantified using a NanoDrop ND-1000 spectrophotometer. cDNA was synthesized by reverse transcription using the PrimeScript RT reagent kit. qRT-PCR was performed using SYBR Premix Ex Taq II on a Lightcycler 96 system (Roche, Basel, Switzerland). Data were analyzed using the $2^{-\Delta\Delta CT}$ method. All results are expressed as the mean ± standard deviation of three independent experiments. Primer sequences were listed in Table S1.

Protein Extraction and Western Blot Analysis: Cells or tissues were lysed with ice-cold lysis buffer (25 mM Tris-HCl, pH 7.4; 100 mM NaF; 50 mM $Na_4P_2O_7$; 10 mM Na_3VO_4; 10 mM EGTA; 10 mM EDTA; 1% NP-40; 10 μg/ml Leupeptin; 10 μg/ml Aprotinin; 2 mM PMSF and 20 nM Okadaic acid). Protein concentration of each sample was determined by BCA assay using the BCA kit according to manufacturer's instructions. The PVDF membranes were incubated with the primary antibodies including anti-TAK1, anti-RASSF9, anti-phospho-RASSF9, anti-c-RAF, anti-phospho-c-RAF, anti-MEK1/2, anti-phospho-MEK1/2, anti-ERK1/2, anti-phospho-ERK1/2, anti-Fos, anti-c-Myc. Mouse anti-β-actin was used as an internal control. Membranes were then incubated with the second HRP-conjugated antibodies. Finally, western blot images protein were visualized by incubating with an enhanced chemilluminescense detection reagent (Roche) and quantified the bands with the ImageJ software.

Colony Formation Assay: Transfected cells were seeded into 6-well plates at 300 cells per well and make sure the indicated cells were in the exponential growth phase. After two

week-incubation at 37°C, cells were fixed with 4% paraformaldehyde (PFA) and stained with 0.1% crystal violet for about 40 min. Finally, the colonies were counted and photographed. Only colonies with diameter more than 40 μm were counted and compared.

CCK-8 Assay: Cell viability was measured by CCK-8 assay as the manufacturer's instructions in three independent experiments. Transfected ESCC cells at a density of 5×10^3 cells/well were seeded in 96-well plates with 100 μl complete medium. After cultured for 24 h, 48 h and 72 h at 37°C, 10 μl of the CCK-8 reagent was added into each well and then incubated for another 4 h. The cell viability was then generated on the basis of the absorbance at a wavelength of 450 nm (OD450) by using a Microplate Reader (BioTek, Winooski, VT).

EdU assay: Cell proliferation ability was researched by using EdU assay, transfected ESCC cells (5×10^4 cells/well) were seeded into 24-well plates with coverslips and incubated. After 24 h, 1 × EdU working solution (10 μM) was added to the wells and cultured for another 2 h at 37°C. Then EdU labeled cells were fixed, permeabilized and incubated in Click reaction mixture for 30 min at room temperature in dark. Coverslips were washed with 0.01 M PBS thrice and stained with Hoechst for cell nuclei for 10 min. Finally, cell proliferation was observed under a fluorescent microscope (Zeiss, Oberkochen, Germany).

Flow Cytometry (FCM) Assay: Cells were collected and washed with 0.01% PBS twice. Afterwards, 195 μl binding buffer was added to resuspend the cells, and 5 μl of Annexin V-FITC and 10 μl Propidium Iodide (PI) (Beyotime Institute of Biotechnology, Haimen, China) was added. The staining process was undergone in dark at room temperature for 10-20 min. Then, fluorescence of FITC and PI was detected by FCM (FACSCalibur, BD Bioscience, Franklin Lakes, NJ, USA). Macquit software (BD Bioscience, Franklin Lakes, NJ, USA) was used to analyze the data.

Immunohistochemistry and Immunostaining: 10% formaldehyde-fixed esophageal tumor tissue or normal tissue sections were deparaffinized in xylene (10 min, thrice), then rehydrated in graded alcohols (100%, 100%, 95%, 90%, 80%, and 70%, each for 5 min), and finally

washed with 0.1 M PBS for three times. The slides were submerged in Tris-EDTA buffer (100, 20 min) to antigen retrieval, and cooled down naturally at room temperature. Blocking with 10% goat serum (contained with 0.1% triton-100) for 30 min at 37°C after treated with endogenous peroxidase blocker buffer for 5 min, and followed by incubated in TAK1 antibodies overnight at 4°C. After washing with 0.1 M PBS for 3 time, sections were incubated with HRP-conjugated secondary antibodies for 1 h at 37°C. Then, hematoxylin was used for redyeing, and finally the sections were dehydrated in graded alcohols and transparented in xylene. After staining, observed under a microscope and randomly selected 5 fields in each section (with the magnification of 400X). The scoring criteria (semi-quantitative method) are comprehensive and determined by staining intensity and proportion of positive cells. Staining intensity score: 0 points for non-staining, 1 point for weak (light yellow), 2 points for moderate (yellowish-brown), 3 points for strong (brown). The score for the proportion of positive cells: 0 for ≤ 5%, 1 for 5% - 25%, 2 for 25% - 50%, 3 for 50% - 75%, 4 for > 75%. A final score was multiplied the two scores, 0-4 indicated negative, 4-8 was weakly positive, > 8 was strong positive. Immunostaining procedures were described previously.[1] For quantitative analysis, regions of interest were captured using 20X objective from a random area of the sections. Ki-67 fluorescence intensity was measured in five fields in each section using ImageJ software. Five sections were analyzed per mouse.

Co-Immunoprecipitation and MS/MS Spectrometry: ECA-109 cells were transfected with plasmid expressing *Map3k7*. 36 h post-transfection, cells were subjected to co-immunoprecipitation experiment by using a commercial kit (Thermo Scientific). 200 μg of proteins for each sample were incorporated into 30 μl SDT buffer (4% SDS, 100 mM DTT, 150 mM Tris-HCl, pH 8.0). The detergent, DTT and other low-molecular-weight components were removed using UA buffer (8 M Urea, 150 mM Tris-HCl, pH 8.0) by repeated ultrafiltration (Microcon units, 10 kD). Then 100 μl iodoacetamide (100 mM IAA in UA buffer) was added to block reduced cysteine residues and the samples were incubated for 30 min in darkness. The filters were washed with 100 μl UA buffer three times and then 100 μl 25 mM NH_4HCO_3 buffer twice. Finally, the protein suspensions were digested with 4 μg trypsin (Promega) in 40 μl 25 mM NH_4HCO_3 buffer overnight at 37 °C, and the resulting peptides were collected as a filtrate.

The peptides of each sample were desalted on C18 Cartridges (Empore™ SPE Cartridges C18 (standard density), bed I.D. 7 mm, volume 3 ml, Sigma), concentrated by vacuum centrifugation and reconstituted in 40 µl of 0.1% (v/v) formic acid. The peptide content was estimated by UV light spectral density at 280 nm using an extinctions coefficient of 1.1 of 0.1% (g/l) solution that was calculated on the basis of the frequency of tryptophan and tyrosine in vertebrate proteins. The resulting samples were subjected to LC-MS/MS spectrometry analysis (Shanghai Applied Protein Technology Co., Ltd.). MS/MS spectra were searched using MASCOT engine (Matrix Science, London, UK; version 2.4) against the UniProtKB human (161584 total entries, downloaded 20180105).

Co-immunoprecipitation assay: HEK293 cells were transfected with the plasmid expressing *Map3k7* by lipofectamine. 24 h post-transfection, cells were subjected to co-immunoprecipitation assay with a commercial kit (Thermo Scientific; Prod#26149) by using an anti-TAK1 antibody (Cell Signaling; Cat#5206). The resulting immuno-complex was analyzed by western blot by using an anti-RASSF9 antibody. To eliminate signals of heavy chain, the light chain specific second antibody (Cell Signaling; Cat#58802) was employed.

SP pull-down assay: HEK293 cells were transfected with the plasmid expressing SP-RASSF9 or SP-EV (empty vector). 24 h post-transfection, cells were harvested for preparing total cell lysates, which was then subjected to SP pull-down assay using S-protein agarose (Novagen; Cat. No. 3226253).[2] The eluted samples were denatured by adding 1X Laemmli buffer and boiled at 100°C for 5 min. After cool down to room temperature, the samples were analyzed by western blot.

Reference:

[1] S. Peng, C. Wang, J. Ma, K. Jiang, Y. Jiang, X. Gu, C. Sun, British journal of pharmacology 2018, 175, 631.
[2] R. T. Raines, M. McCormick, T. R. Van Oosbree, R. C. Mierendorf, Methods in enzymology 2000, 326, 362.
